# Pathogenesis and therapeutic strategies for irritable bowel syndrome: an integrated perspective from dynamic balance theory of Chinese medicine

**DOI:** 10.3389/fmed.2026.1823683

**Published:** 2026-08-19

**Authors:** Nanxi Liu, Yahui Huang, Yue Wang, Huixia Qiao

**Affiliations:** 1 The First School of Clinical Medicine, Shaanxi University of Traditional Chinese Medicine, Xianyang, China; 2 Xi'an Hospital of Traditional Chinese Medicine, Xi'an, China

**Keywords:** complementary therapy, heart-liver-spleen theory, irritable bowel syndrome, pathophysiology, traditional Chinese medicine

## Abstract

**Background:**

Irritable bowel syndrome (IBS) is a common disorder of gut-brain interaction characterized by recurrent abdominal pain and altered bowel habits, resulting in a substantial impact on quality of life. Owing to the multifactorial nature of IBS and the limitations of current treatment options, Traditional Chinese Medicine (TCM) has increasingly been investigated as a complementary therapeutic approach.

**Aim:**

This narrative review examines the pathogenesis of IBS from the perspective of TCM, with particular emphasis on the interrelationships among the heart, liver, and spleen within the framework of interpromotion and interrestraint theory. It also summarizes representative TCM-based intervention strategies and discusses their potential conceptual links to contemporary biomedical mechanisms.

**Methods:**

A narrative review approach was adopted. Relevant literature concerning IBS pathogenesis, TCM theories, and TCM-based interventions was identified through searches of major scientific databases. Findings from experimental and clinical studies were synthesized qualitatively to provide an overview of current evidence and research trends.

**Results:**

Current evidence suggests that TCM interventions may influence multiple biological processes implicated in IBS, including brain-gut axis signaling, gastrointestinal motility, gut microbiota composition, intestinal barrier function, immune regulation, and visceral sensitivity. Emerging studies also suggest potential conceptual associations between traditional TCM concepts and modern pathophysiological mechanisms; however, these relationships remain hypothesis-generating and require further experimental validation. However, interpretation of the evidence is limited by heterogeneity in study design, syndrome differentiation, intervention protocols, and outcome assessment.

**Conclusion:**

TCM provides a complementary framework for understanding and managing IBS and may offer therapeutic benefits through multidimensional regulatory effects. Nevertheless, the overall quality of available evidence remains variable, and many mechanistic findings are derived primarily from preclinical studies. Further high-quality clinical research, standardized outcome measures, and biomarker-based investigations are needed to clarify the clinical value and biological basis of TCM interventions in IBS.

## Highlights

- Proposes a novel TCM framework explaining IBS pathophysiology via heart-liver-spleen system interactions based on the Five-Phase theory.
- Bridges TCM and modern mechanisms by connecting liver-spleen dysfunction and visceral hypersensitivity to the brain-gut-microbiota axis.
- Synthesizes evidence for multi-targeted TCM therapies (e.g., herbal formulas, acupuncture) that modulate gut microbiota, reduce visceral hypersensitivity, and regulate motility.

## 1 Introduction

Irritable bowel syndrome (IBS) is a common functional gastrointestinal disorder characterized by recurrent abdominal pain and altered bowel habits, significantly impairing patients’ quality of life. Epidemiological studies estimate a global prevalence of approximately 11.0%, with about 6.5% in China (1). IBS is more frequently diagnosed in women than men, particularly among young, educated, and affluent populations (2–4). Large-scale studies using Rome IV criteria have reported similar prevalence in Europe and the United States, and relatively lower rates in Asia and Australia (5). Individuals with higher levels of anxiety or depression often experience greater impairment in work and daily activities (6), and healthcare costs in the United States exceed $1 billion annually (7), highlighting the urgent need for effective interventions.

The pathophysiology of IBS is multifactorial and not fully understood. Key mechanisms include dysregulation of the brain-gut axis, altered gastrointestinal motility, visceral hypersensitivity, immune dysfunction, and gut microbiota imbalance (1). These processes underlie symptom development and represent potential therapeutic targets. Current medical treatments primarily aim to relieve symptoms through analgesics, motility-modifying agents, antidiarrheals, and psychological interventions (8). Although these approaches provide benefit for many patients, responses are highly variable, and some individuals experience persistent or recurrent symptoms (9), underscoring the need for complementary strategies.

Traditional Chinese Medicine (TCM) offers a holistic approach that emphasizes pattern-based diagnosis (syndrome differentiation) and the dynamic interactions among the heart, liver, and spleen. These relationships are explained by the theory of interpromotion and interrestraint, a conceptual framework describing mutually supporting and regulating relationships among organ systems within TCM theory. Within the TCM framework, these relationships are used to interpret symptom patterns and guide therapeutic strategies for IBS. According to TCM theory, IBS manifestations have traditionally been interpreted as being associated with liver-spleen disharmony, spleen-stomach weakness, or dual deficiency of the heart and spleen, while emotional stress, dietary irregularities, and climatic influences are considered important contributing factors. Clinical interventions are based on Wu Xing (Five Phases) theory, a TCM framework describing the relationships among wood, fire, earth, metal, and water corresponding to the liver, heart, spleen, lung, and kidney, respectively. These interventions include herbal medicine and acupuncture. They have shown efficacy in alleviating symptoms and improving quality of life (10).

This review first summarizes current understanding of IBS pathophysiology based on contemporary biomedical evidence and subsequently discusses how the heart-liver-spleen framework of TCM may provide a complementary perspective for interpreting clinical manifestations and guiding intervention strategies. Representative TCM-based approaches and the available evidence supporting their use are also reviewed. By examining potential areas of conceptual convergence between traditional concepts and modern biomedical findings, this review aims to provide an integrated perspective on the potential role of TCM in IBS management.

## 2 Modern medical recognitions of IBS

### 2.1 Classification, epidemiological characteristics, and clinical manifestations of IBS

The diagnosis of IBS primarily relies on clinical symptom assessment, as no specific biomarkers are currently available. According to the Rome IV diagnostic criteria (11), IBS is classified into four subtypes based on the Bristol Stool Form Scale: diarrhea-predominant IBS (IBS-D), constipation-predominant IBS (IBS-C), mixed IBS (IBS-M), unclassified IBS (IBS-U). Among these, IBS-D is the most prevalent subtype. Patients typically experience recurrent abdominal pain at least once per week, which is commonly associated with changes in stool frequency or form and is often relieved or exacerbated by defecation. This symptom-based classification provides a fundamental framework for the clinical diagnosis and management of IBS.

**Figure 1:**
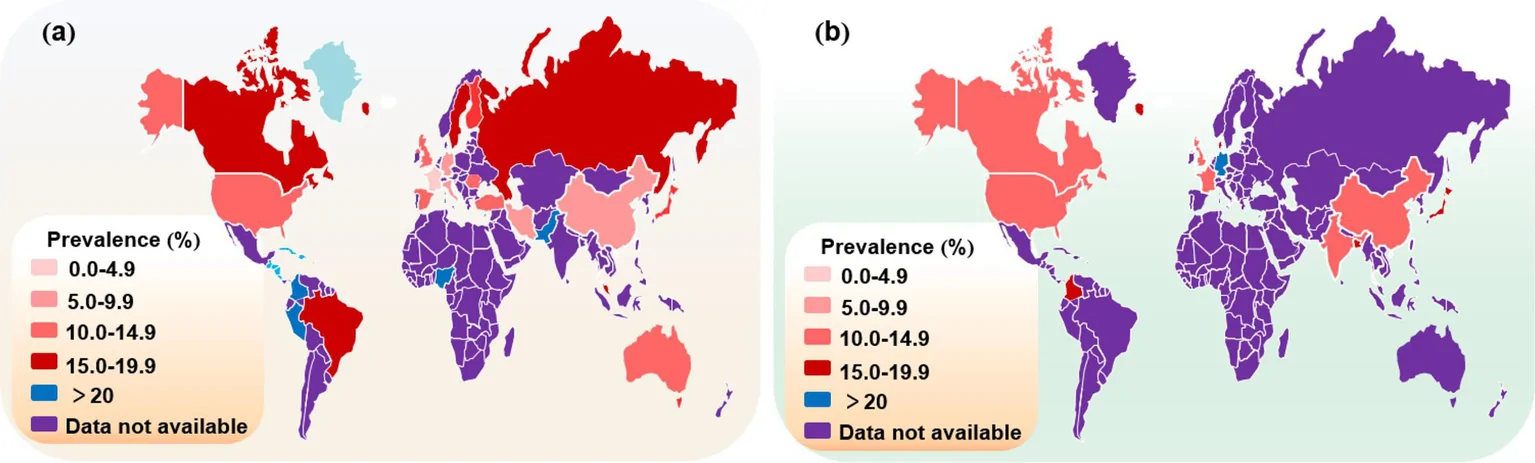
Global prevalence of IBS: **(a)** Prevalence of IBS by country in the Rome I/Rome II criteria; **(b)** Prevalence of IBS by country in the Rome III criteria (112).

At the same time, there is significant global variability in the prevalence of IBS. A 2020 study estimated the prevalence of functional gastrointestinal disorders across multiple countries using Rome criteria. Data were collected through online surveys in Canada and the United Kingdom, as shown in Figure 1. Figure 1a illustrates IBS prevalence based on the Rome I and II criteria. North America, Russia (Asia), and Eastern Africa show high prevalence rates (15 ~ 19.9%), while China and parts of Europe exhibit moderate rates (5.0 ~ 9.9%). Figure 1b presents IBS prevalence based on the Rome III criteria, showing rates of 10.0 ~ 14.9% in regions such as North and South America, and specific areas of Asia (e.g., China and India). As shown, IBS prevalence varies markedly across geographical regions. High-income regions, including North America and Europe, generally report higher prevalence, likely reflecting well-developed healthcare systems, broader adoption of diagnostic criteria, and more comprehensive survey coverage. In contrast, lower-middle- and low-income regions, particularly in parts of Africa and Asia, tend to report lower prevalence, largely due to limited healthcare access and insufficient epidemiological data.

The primary clinical manifestations of IBS include abdominal discomfort or recurrent pain, typically relieved by defecation and associated with changes in stool pattern or frequency. IBS is commonly accompanied by emotional disturbances, such as anxiety and depression (11), as well as extraintestinal symptoms including heartburn, dyspepsia, chest pain, bloating, defecatory urgency, incomplete evacuation, chronic pelvic pain, fibromyalgia, and migraine (12). As a chronic, intermittent, and recurrent functional gastrointestinal disorder without identifiable organic pathology, IBS often coexists with other functional or structural conditions, such as gastroesophageal reflux disease (GERD) and functional dyspepsia, and typically persists for at least 6 months.

### 2.2 The pathogenesis of IBS

**Figure 2:**
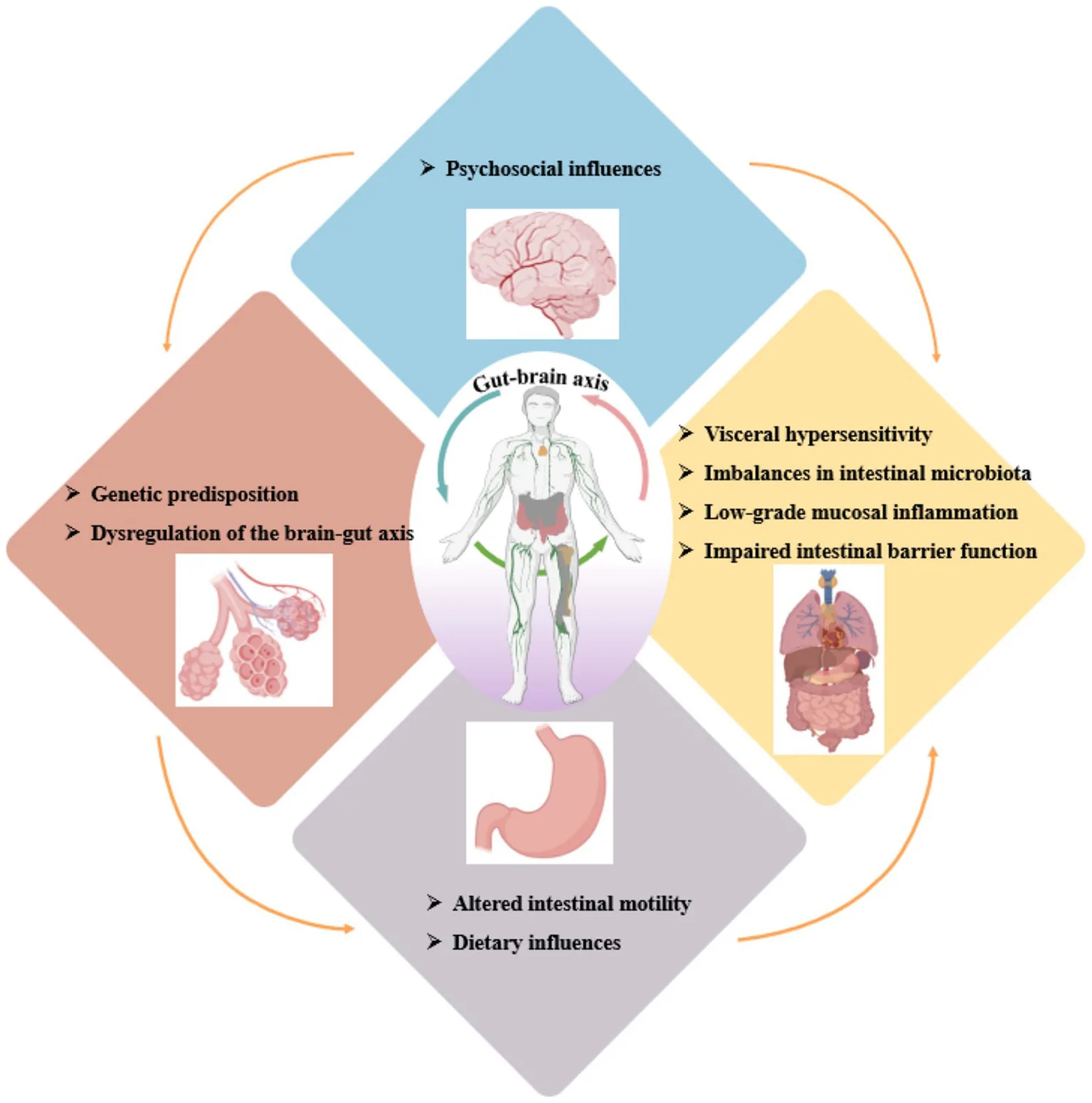
Pathogenesis of IBS.

The pathogenesis of IBS remains incompletely understood and may stem from multifactorial interactions involving a combination of multiple factors, as shown in Figure 2. Evidence suggests that key mechanisms involved in IBS pathogenesis include abnormal central nervous system (CNS) activity, psychosocial influences, genetic predisposition, dysregulation of the brain-gut axis (13, 14), altered intestinal motility (15), visceral hypersensitivity, imbalances in intestinal microbiota, low-grade mucosal inflammation (16), impaired intestinal barrier function, dietary influences, and so on. These mechanisms above lead to IBS symptoms.

#### 2.2.1 Abnormal brain-gut axis regulation

Traditionally, the pathogenesis of IBS has been closely associated with dysfunction of the gut-brain axis, largely due to its strong links with CNS alterations such as anxiety and depression. A meta-analysis by Zamani et al. (17) reported prevalence rates of anxiety and depression among IBS patients of 39.1 and 28.8%, respectively, approximately threefold higher than in healthy controls. The gut-brain axis is a bidirectional neurohumoral communication system involving the autonomic nervous system, the hypothalamic–pituitary–adrenal (HPA) axis, and the gut microbiota, which together regulate gastrointestinal function and microbial homeostasis (18, 19). Recent studies increasingly conceptualize IBS-related pain as a form of nociplastic pain, involving altered central pain processing and dysregulated brain-gut communication rather than solely peripheral gastrointestinal abnormalities (20, 21).

**Figure 3:**
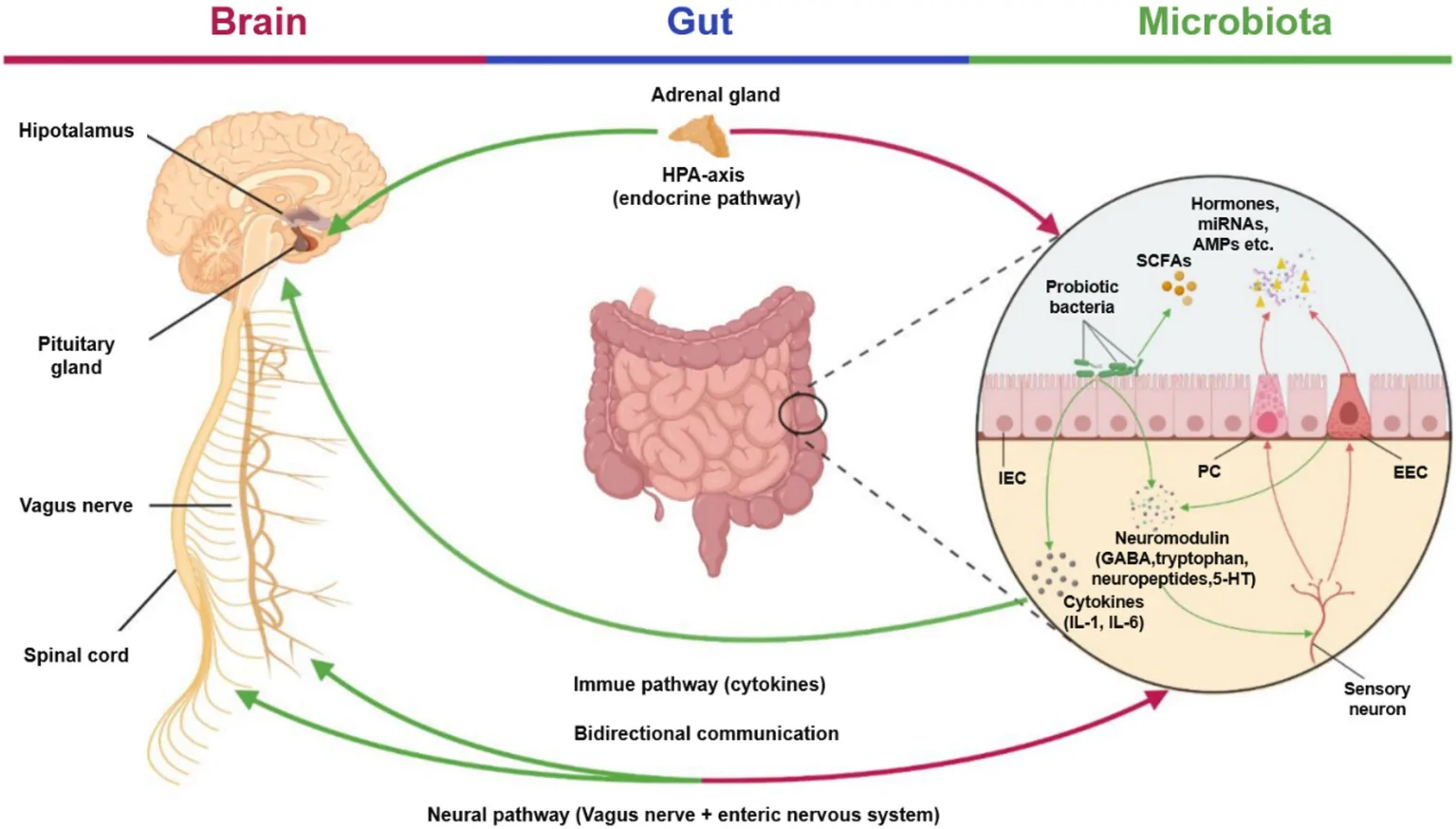
Microbial community-gut-brain axis communication routes (113). IEC, intestinal epithelial cells; PC, Paneth cell; IL, interleukin; GABA, *γ*-aminobutyric acid; 5-HT (5-hydroxytryptamine), Serotonin; AMP, antimicrobial peptide.

Figure 3 illustrates the communication pathways between gut microbiota and the brain, including the vagus nerve, cytokines, gut-derived neuropeptides, sensory neurons, tryptophan (Trp), and short-chain fatty acids (SCFAs) (22). Conversely, signaling molecules secreted by enteroendocrine cells (EECs) mediate communication from gut to brain. Additionally, gut microorganisms can uptake microRNAs (miRNAs), influencing microbial activity (23). Among these pathways, the endocrine route, mainly involving the HPA axis and EECs, is extensively studied. Neural pathways such as the vagus nerve and enteric nervous system, along with immune signaling via cytokines, also contribute. Together, these pathways maintain gut-brain homeostasis, playing a crucial role in IBS pathogenesis.

#### 2.2.2 Visceral hypersensitivity

A study of 407 patients highlighted visceral hypersensitivity, colonic transport issues, and psychological factors as key contributors to symptoms and quality of life. Visceral hypersensitivity links to specific brain areas (24). Anxiety and depression increase pain sensitivity and inflammation in IBS patients (25). In animal models, depression exacerbates IBS-like symptoms more than Post-Traumatic Stress Disorder or healthy controls (26).

A Rome Foundation survey found 55.5% of functional bowel disorder patients have psychological distress or somatic symptoms (27), underscoring the need for multidisciplinary IBS treatment. Visceral hypersensitivity may involve mast cell (MC) abnormalities activating enteric neurons (28). Gut dysbiosis may influence mast cell function, but its precise role in IBS is unclear. Further research on MC-neural interactions is needed to develop targeted therapies.

#### 2.2.3 Psychological factors

Psychological factors or gastrointestinal infections may induce structural and functional alterations in the enteric nervous system. This system regulates gastrointestinal motility, sensation, mucosal barrier integrity, and secretory responses via specific signaling pathways (29). In IBS patients, microbiome composition differs between those with and without psychological complication (30). Studies have demonstrated that IBS patients exhibit reduced psychological and physiological resilience to stress, potentially increasing their vulnerability and impairing recovery (31).

Psychological disturbances in IBS have been associated with alterations in serotonergic and neuroendocrine signaling, which may partially correspond to heart-related functions in TCM. Mood changes in IBS relate to brain function alterations; for instance, IBS patients with alexithymia show distinct right insula responses to rectal distention (32). CNS neurotransmitters regulate gastrointestinal sensory, motor, and secretory functions, underpinning the brain-gut axis (33). Heart rate variability (HRV), reflecting autonomic nervous system (ANS) balance, indicates parasympathetic activity and stress adaptability (34). The low-frequency to high-frequency (LF/HF) ratio measures autonomic balance, correlating positively with IBS symptoms, anxiety, depression, and stress (35).

#### 2.2.4 Gut dysbiosis

The gut microbiota plays a central role in the pathogenesis of IBS. Microbial dysbiosis associated with IBS affects gut motility, visceral sensitivity, gut-brain axis signaling, immune activation, and mucosal permeability. Gut microbiota dysregulation increases intestinal permeability. This may predispose individuals to low-grade mucosal inflammation and immune activation (36).

IBS is characterized by altered microbiota composition, impaired mucosal barrier integrity, and low-grade inflammation. Barrier dysfunction increases exposure to luminal antigens, contributing to abdominal pain and altered bowel habits, primarily through immune activation and visceral hypersensitivity (37, 38). Emerging evidence further links gut dysbiosis and metabolic disturbances with the development and progression of IBS-D (39).

Overall, the gut microbiota is essential for gastrointestinal homeostasis, immune regulation, nutrient absorption, and host metabolism, and contributes to IBS pathogenesis by modulating motility, sensitivity, inflammation, and metabolic processes.

## 3 TCM perspectives on IBS

### 3.1 TCM attribution and etiology of IBS

IBS has no specific disease category in TCM and is generally classified as “constipation,” “abdominal pain,” “diarrhea,” or “depressed syndrome.” Its pathogenesis is attributed to six climatic exopathogens, emotional disturbances, dietary irregularities, constitutional deficiency, and overstrain, leading to dysfunction of the liver, spleen, stomach, heart, and gallbladder, and resulting in impaired gastrointestinal motility and mixed deficiency excess patterns with cold and heat intermingling.

Seasonal pathogenic factors, particularly dampness during humid-earth periods, are considered important triggers that further impair digestive function. Emotional stress and internal disharmony may aggravate organ dysfunction, leading to abdominal pain, distension, diarrhea, and other gastrointestinal symptoms.

In summary, IBS is characterized by complex interactions between external pathogenic factors and internal organ dysfunction, reflecting the holistic view of TCM and supporting syndrome-based therapeutic strategies for symptom relief and improved quality of life (40).

### 3.2 Heart-liver-spleen interactions and IBS pathogenesis

#### 3.2.1 Heart dysfunction affecting liver-spleen systems

The heart-spleen relationship is traditionally associated with blood production, circulation, and digestive function. According to TCM theory, heart deficiency may impair spleen function, thereby contributing to digestive disorders such as diarrhea and constipation.

The heart-liver relationship is closely linked to emotional regulation. Emotional disturbances, including anxiety and depression, have been associated with IBS and may affect gastrointestinal function through the brain-gut axis (36, 41). Altered 5-HT signaling has been implicated in this process and may contribute to abnormal gastrointestinal motility, secretion, and visceral sensitivity, resulting in symptoms such as abdominal pain and diarrhea (42).

#### 3.2.2 Liver dysfunction affecting heart-spleen systems

In TCM, the liver regulates the smooth flow of energy and blood. The spleen and stomach are responsible for digestion and nutrient absorption. The heart is associated with mental and emotional activities. It is involved in psychological regulation. The interaction between the liver, heart, and spleen is important for physiological balance.

Emotional stress can affect heart function. This may lead to mood changes and emotional imbalance. These changes can further impair liver function. As a result, spleen and stomach function may be disrupted. Gastrointestinal motility may change. Abdominal pain and bowel dysfunction may occur in IBS patients (43).

IBS is closely related to psychological stress. Stress can activate the hypothalamic–pituitary–adrenal axis. This leads to emotional disorders and gastrointestinal symptoms (44, 45).

**Figure 4:**
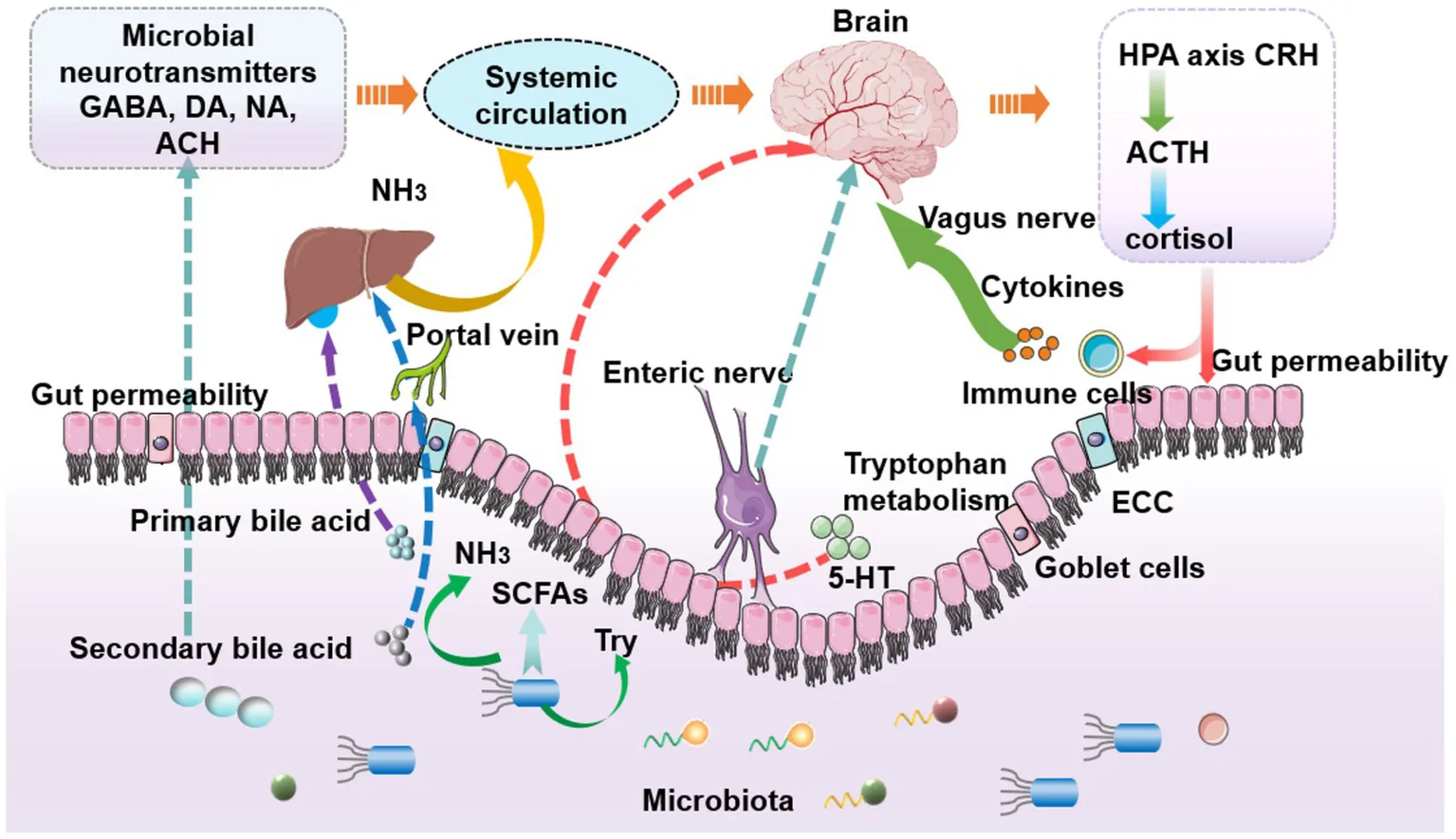
Various complex bidirectional information interaction mechanisms in the microbiota-intestinal-liver-brain axis (48). DA, dopamine; NA, norepinephrine; Ach, acetylcholine; CRH, corticotropin-releasing hormone; ACTH, adrenocorticotropic hormone; ECC, enteric chromaffin cells.

The brain-gut axis is a bidirectional communication system between the central nervous system and the gastrointestinal tract. It involves neural, endocrine, and immune pathways. It regulates both brain and gut function. The relationship is shown in Figure 4.

In IBS-D, about 25% of patients have impaired bile acid absorption or idiopathic bile acid diarrhea. Increased bile acids can promote colonic secretion, increase permeability, and enhance motility. These changes contribute to IBS-D symptoms (46).

The liver-intestine axis is a communication system between the liver and the gut. It is mediated through portal circulation and bile acid signaling. It helps maintain intestinal homeostasis (47, 48).

From both TCM and modern perspectives, dysfunction of the liver may affect the heart and spleen. This can lead to emotional disturbance, abdominal pain, bloating, and altered bowel habits in IBS (49, 50).

#### 3.2.3 Spleen dysfunction affecting heart-liver systems

In the Five Phases theory, the spleen corresponds to the earth phase and, together with the stomach, forms the digestive core. It is considered essential for postnatal health, governing the transport and transformation of nutrients and fluids to sustain metabolism and blood formation (51).

The spleen is essential for maintaining the physiological functions of the liver, heart, lung, and kidney, thereby supporting systemic homeostasis. Spleen deficiency impairs transformation and transportation, resulting in reduced production of essential nutrients and blood. This may disrupt liver function, and prolonged deficiency may further impair cardiac function due to insufficient nourishment, ultimately leading to systemic imbalance.

#### 3.2.4 Role of generation-restriction-counteraction imbalance in IBS pathogenesis

TCM emphasizes holism and syndrome differentiation. According to the Five Phases theory, Zang-Fu organs are the internal functional organ systems in TCM. According to the Five Phases Theory, the zang-fu organs are interconnected through mutual promotion and mutual restraint. The zang-fu organs are internal organs with their associated functions in TCM. Zang refers to yin organs of solid tissues, while fu refers to yang organs of hollow cavities. These interactions maintain physiological balance. Disruption of these relationships may affect multiple organ systems.

In this framework, the heart, liver, and spleen are considered key organs involved in emotional regulation, stress adaptation, and digestive function. Dysfunction of these systems has been proposed to contribute to IBS pathogenesis. Accordingly, TCM interventions aim to restore functional balance through individualized and holistic treatment strategies.

Although the heart-liver-spleen framework originates from TCM theory, emerging evidence suggests potential biological correlates. For example, heart-related functions associated with emotional regulation may involve neuroendocrine and CNS signaling pathways; liver-related dysfunction may be linked to stress responses, autonomic regulation, and brain-gut communication; whereas spleen-related dysfunction has been associated with alterations in intestinal barrier integrity, gut microbiota composition, and metabolic homeostasis. These observations provide a potential bridge between classical TCM concepts and contemporary molecular mechanisms, although direct causal relationships remain to be established (52, 53). The manifestations of heart-liver-spleen imbalance may differ across IBS subtypes, with IBS-D commonly characterized by liver overacting on the spleen, IBS-C by impaired spleen transportation and intestinal dryness, and IBS-M by dynamic fluctuations between these patterns (as summarized in Tables 1, 2).

**Table 1:** Conceptual interpretation of TCM theoretical framework in relation to contemporary biomedical understanding of IBS.

TCM concept	Proposed biomedical correlate
Heart dysfunction	Emotional regulation, CNS signaling
Liver stagnation	Stress response, autonomic dysfunction, brain–gut axis
Spleen deficiency	Gut microbiota dysbiosis, barrier dysfunction, metabolic abnormalities
Heart-liver-spleen imbalance	Multisystem dysregulation involving neuroendocrine, immune, and microbiota pathways

The proposed correspondences between TCM concepts and contemporary biomedical mechanisms represent conceptual and hypothesis-generating interpretations of potential relationships between TCM theoretical frameworks and biomedical understanding of IBS. These associations should not be interpreted as direct one-to-one biological equivalencies or experimentally validated mechanistic relationships.

**Table 2:** Proposed conceptual associations among IBS subtypes, core mechanisms, and TCM syndrome frameworks.

IBS subtype	Core mechanisms	Representative TCM syndromes	Key therapeutic principles
IBS-D	Brain-gut axis dysregulation; altered gastrointestinal motility; visceral hypersensitivity; low-grade intestinal inflammation; gut microbiota imbalance	Spleen deficiency, spleen-kidney yang deficiency, liver-stagnation with spleen deficiency	Invigorate spleen, warm yang, regulate liver, strengthen intestinal barrier, modulate gut microbiota
IBS-C	Slow intestinal transit; impaired gut motility; phlegm-damp accumulation; visceral hypersensitivity	Phlegm-turbidity obstructing middle jiao; intestinal dryness; spleen-stomach deficiency	Promote intestinal motility, resolve phlegm, moisten intestines, strengthen spleen, clear heat
IBS-M	Dysregulated motility; visceral hypersensitivity; intestinal inflammation; microbiota imbalance	Liver-spleen disharmony; spleen-stomach deficiency	Harmonize liver and spleen, regulate bowel movements, reduce inflammation, modulate microbiota
IBS-U	Variable motility changes; mild inflammation; altered gut-brain axis	Mild spleen or liver disharmony	Individualized pattern-based treatment: support spleen, regulate liver, resolve mild dampness or stagnation

IBS subtypes follow the Rome IV classification. Core mechanisms summarize current biomedical understanding of IBS, including brain–gut axis, microbiota, motility, inflammation, and visceral hypersensitivity. TCM syndromes are described according to major patterns reported in classical texts and contemporary studies. These proposed correspondences represent conceptual and hypothesis-generating interpretations rather than experimentally validated mechanistic relationships or direct biological equivalencies.

## 4 Western medical treatment methods

### 4.1 Pharmacological treatment

Current management of IBS is based on a combination of dietary, behavioral, and pharmacological interventions tailored to symptom subtype and disease severity. Guideline-supported approaches include dietary modification, such as a low fermentable oligosaccharides, disaccharides, monosaccharides, and polyols (FODMAP) diet (LFD), psychological therapies targeting the brain-gut axis, and pharmacological treatments for symptom relief (50, 54).

For IBS-D, commonly used therapies include loperamide, rifaximin, bile acid sequestrants, and other symptom-directed treatments. For IBS-C, therapeutic options include osmotic laxatives, secretagogues, and prokinetic agents. In addition, neuromodulators, including tricyclic antidepressants and selective serotonin reuptake inhibitors, may be considered in selected patients, particularly when abdominal pain or psychological comorbidities are present (50).

Although these therapies can provide meaningful symptom improvement for many patients, treatment responses are often heterogeneous because of the multifactorial nature of IBS. Some medications may be associated with adverse effects, and not all patients achieve sustained symptom control. Consequently, treatment frequently requires individualized adjustment and a multidisciplinary management strategy. Ongoing research continues to explore novel therapeutic approaches aimed at improving clinical outcomes and addressing the complex pathophysiology of IBS.

### 4.2 Dietary therapies

Professor Garg et al. (55) proposed the Fiber, Elevation, Exercise, Dietary modification (FEED) approach for managing IBS-D. It includes 25 g of psyllium fiber and 500 mL of water daily, elevating the feet, and performing abdominal exercises on the toilet. This helps alleviate IBS-D symptoms. In contrast, foods like lactose, sorbitol, fructose, xylitol, mannitol, fats, alcohol, insoluble fiber, and carbonated beverages can worsen pain, gas, and bloating, and should be avoided (56). FEED Therapy provides a non-pharmacological, sustainable management approach through fiber, posture, exercise, and diet. Its focus on bowel posture and personalized diet is highly relevant for patients with functional gastrointestinal disorders or sedentary lifestyles.

The 2021 guidelines by the British Society of Gastroenterology (50), updated by the American College of Gastroenterology (ACG) in 2022, emphasize the importance of dietary counseling as a primary treatment option. Soluble fiber or psyllium is more effective than bran for patients with abdominal pain and IBS discomfort (57). Patients with IBS-D benefit more from the LFD than those with IBS-C, while fiber diets, such as psyllium, are more effective for IBS-C patients (58).

**Figure 5:**
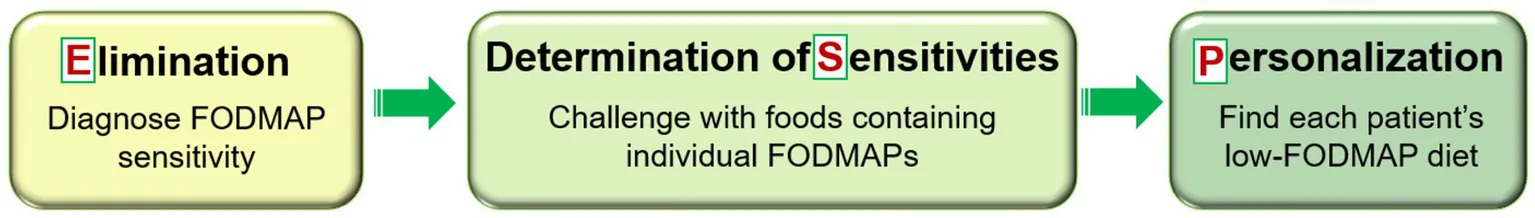
The three stages of a LFD are elimination, determining sensitivity, and personalization.

The LFD consists of three phases, represented by the acronym ESP: the Elimination phase, the Reintroduction phase (which involves reintroducing FODMAP-containing foods to assess individual sensitivity), and the Personalization phase. The process is shown in Figure 5.

LFD effectively relieve symptoms, but concerns have been raised about their impact on gut microbiota composition, with long-term effects unclear (59). McIntosh et al. (60) found that the LFD increased the abundance of *Actinobacteria*, *Thick-walled*, and *Clostridium phyla*, while the high-FODMAP diet reduced *gas-producing bacteria*. FODMAPs, poorly absorbed in the small intestine, are fermented by gut microbiota into SCFAs, leading to gas production, increased osmotic pressure, accelerated gastrointestinal transit, and luminal dilation (61). Breath tests for glucose, lactulose, and other sugars in IBS patients suggest small intestinal bacterial overgrowth, with antimicrobial therapy potentially beneficial for alleviating symptoms like bloating (62).

### 4.3 Psychological therapies

Long-term treatment can impose financial and psychological burdens. Patients unresponsive to conventional therapies should be further evaluated and consider psychotherapy or second-line treatments. A meta-analysis by Weerts et al. (63) (2020) found psychotherapy effective for IBS.

**Figure 6:**
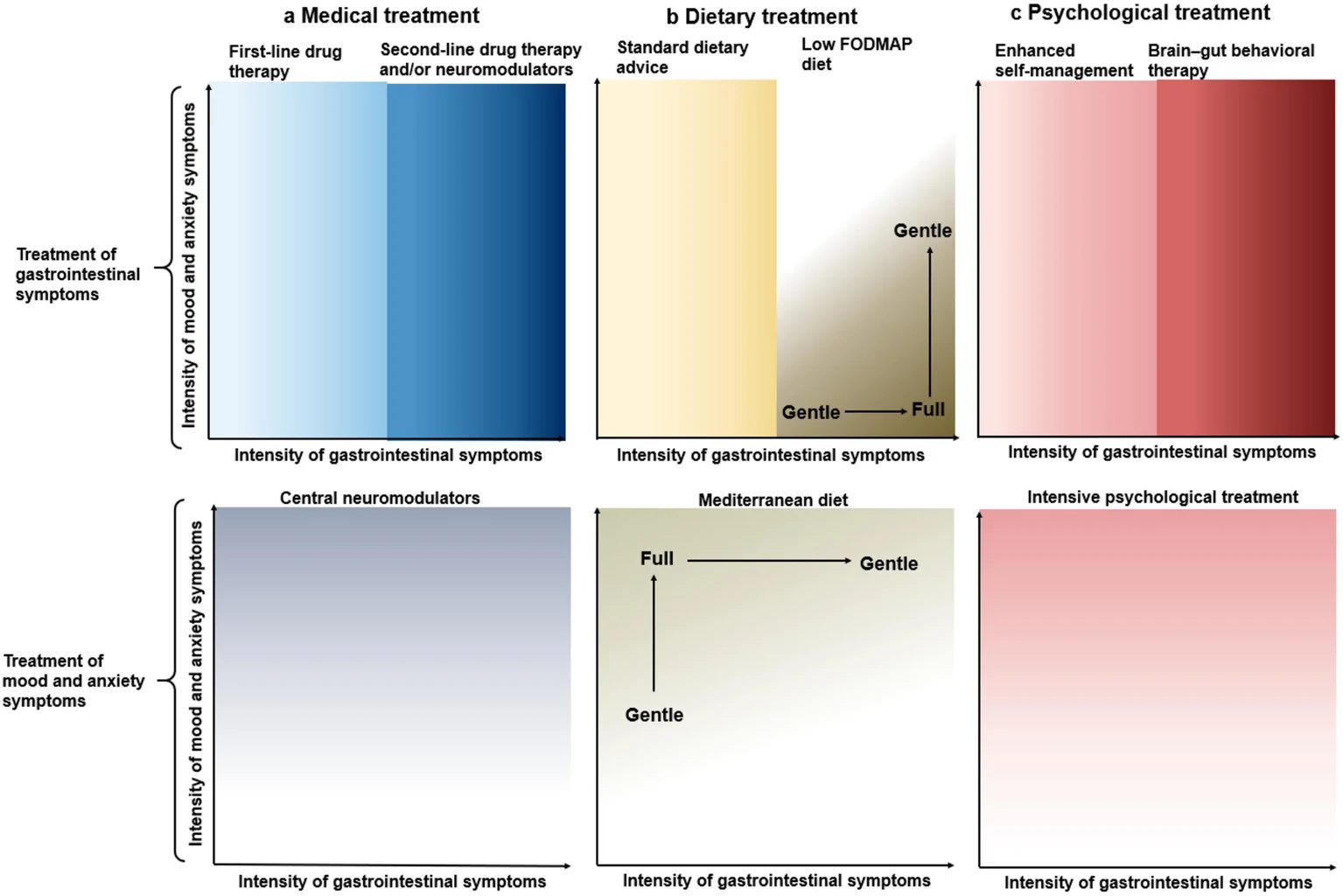
An integrated approach to treating gastrointestinal symptoms: a multilevel strategy involving pharmacological, dietary, and psychological interventions (114).

The microbiome composition differs between IBS patients with and without psychological comorbidities. Therefore, a combination of treatments is recommended to improve IBS symptoms (30). Figure 6 presents an integrated treatment approach for gastrointestinal symptoms, focusing on three areas: drug, diet, and psychology. If medication is the initial treatment, dietary therapy provides guidance on dietary management. Psychotherapy can also improve symptoms by enhancing self-management and brain-gut behavioral therapy. Additionally, gastrointestinal and mood-anxiety symptoms exhibit dynamic progression, varying from mild to severe and vice versa. The approach also includes central neuromodulators, the Mediterranean Diet, and intensive psychotherapy. Overall, a multilevel, comprehensive treatment approach, including pharmacological, dietary, and psychological interventions, may effectively alleviate gastrointestinal symptoms and associated emotional anxiety.

### 4.4 Fecal microbiota transplantation

Fecal microbiota transplantation (FMT) may benefit IBS patients by correcting gut dysbiosis (64), but current randomized controlled trials do not show significant improvement in overall IBS symptoms. While FMT has been effective in treating *Clostridioides* difficile infection (CDI) (65), studies on FMT for IBS are limited, and results remain controversial. This highlights the need for careful consideration and judicious use of medications in clinical practice.

In summary, selecting drugs for IBS-D treatment is challenging, requiring a balance of efficacy, safety, and side effects. It is essential to explore safer and more effective treatments.

## 5 Clinical intervention strategies in TCM

### 5.1 Pattern identification and treatment

Given the heterogeneity of treatment responses in IBS, there is growing interest in complementary and integrative therapeutic approaches, including herbal medicine (66). TCM applies the principles of syndrome differentiation and individualized treatment, taking into account the patient’s clinical manifestations, constitution, and underlying pathogenic patterns. Several clinical studies and meta-analyses have suggested that TCM interventions may improve IBS-related symptoms and quality of life in some patients, particularly those with IBS-D; however, the quality of evidence varies, and further well-designed studies are needed to confirm these findings (67–69). As an important component of TCM theory, TCM emphasizes the maintenance of physiological balance and harmony. Within this framework, IBS is often interpreted as a manifestation of internal imbalance, and therapeutic strategies are directed toward restoring functional equilibrium (70).

**Figure 7:**
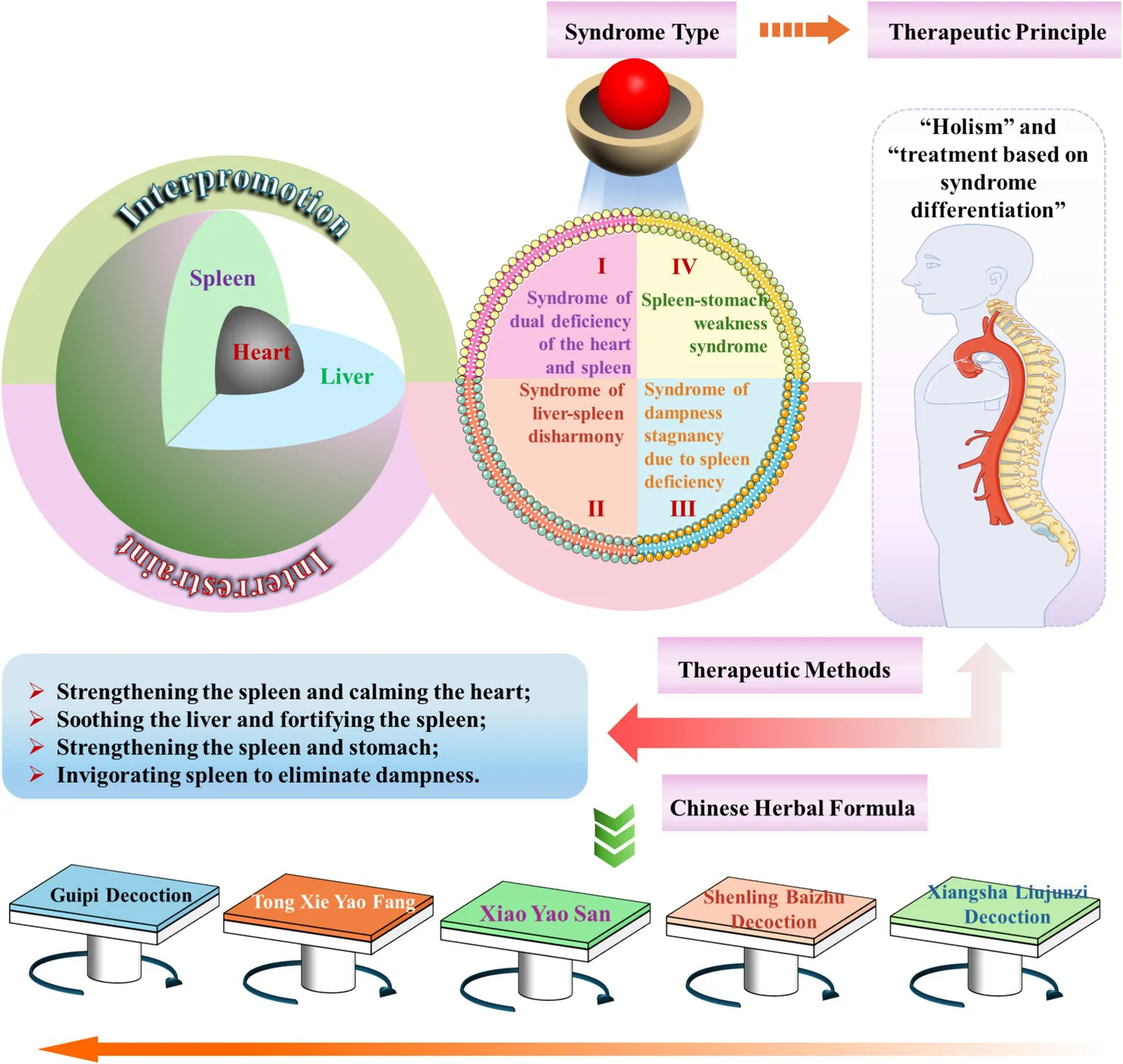
Framework for pattern differentiation and treatment of IBS involving the heart, liver, and spleen.

Guided by the aforementioned theory, Figure 7 presents a diagnostic and treatment framework for IBS based on heart-liver-spleen pattern differentiation, which is a system that classifies patients into functional imbalance subtypes. This framework encompasses several common patterns, including heart-spleen deficiency, dampness stagnancy due to spleen deficiency, spleen-stomach deficiency, and liver-spleen disharmony. Following holistic principles of TCM, therapeutic strategies focus on strengthening the spleen and calming the heart, eliminating dampness, and soothing the liver. Core formulae include Guipi Decoction, Shenling Baizhu Decoction, Xiangsha Liujunzi Decoction, Tong Xie Yao Fang (TXYF), and Xiao Yao San (XYS).

#### 5.1.1 Treating IBS from the heart

Psychological factors closely associate with gastrointestinal function. Mental states directly affect gut motility and digestive secretions, causing constipation. In TCM, this condition is attributed to mental restlessness. Treatment focuses on tranquilizing the mind and moistening the intestines. Treatment options include individual herbal ingredients and modified forms of the classic formula Tianwang Buxin Dan (TWBXD). The herbal ingredients are *Platycladus orientalis* (L.) Franco, *Ziziphus jujuba* Mill., *Ophiopogon japonicus* (Thunb.) Ker Gawl., and *Scrophularia ningpoensis* Hemsl. (Supplementary Tables S1, S2 in the Supplementary material). TWBXD is also known as the Heavenly Emperor Heart-Tonifying Pill. This formula is traditionally used for insomnia and palpitations. These treatments collectively regulate mental activity. At the same time, they relieve constipation by moistening the intestines. The gut-brain-microbiome axis may explain these dual effects. For instance, vagal activity supports intestinal homeostasis through anti-inflammatory mechanisms (71).

Intestinal microbiota is closely associated with psychiatric disorders through bidirectional interactions along the gut-brain axis (72). Emerging evidence suggests that TWBXD may modulate gut microbiota composition and regulate brain-gut signaling pathways, thereby influencing neurotransmitter systems, including 5-HT, and contributing to the improvement of gastrointestinal and emotional symptoms (73). TCM also introduces the “Heart-Spleen Interaction Theory” theory, which emphasizes the mutual dependence of mental activity and digestive function. Guipi Decoction consists of *Astragalus mongholicus* Bunge, *Panax ginseng* C.A.Mey., *Atractylodes macrocephala* Koidz., *Poria cocos* (Schw.) Wolf., *Angelica sinensis* (Oliv.) Diels, *Dimocarpus longan* Lour., *Ziziphus spinosa* Lam (Suanzaoren), *Polygala tenuifolia* Willd., *Dolomiaea costus* (Falc.) Kasana & A.K.Pandey, *Glycyrrhiza glabra* L., *Zingiberis Rhizoma Recens* (Shengjiang) and *Ziziphus jujuba* Mill.(Dazao), and is suitable for the syndrome of dual deficiency of the heart and spleen, and the syndrome of spleen failing to manage blood. Psychological disturbances are increasingly recognized as important contributors to IBS-D pathogenesis through alterations in brain-gut interactions. Accordingly, TCM therapies that emphasize both gastrointestinal regulation and emotional balance may offer a holistic approach to symptom management. This concept is consistent with the traditional principle of simultaneously regulating the spleen and mind, which aligns with current understanding of the brain-gut axis in IBS-D.

#### 5.1.2 Treating IBS from the liver

In TCM, IBS is associated with emotional stress and irregular eating. Its pathogenesis involves liver stagnation, spleen deficiency, and internal dampness. Liver stagnation may reflect stress-related brain-gut axis and neuroendocrine dysregulation, which can be assessed using biomarkers such as 5-HT, vasoactive intestinal peptide (VIP), and somatostatin (SS) (74, 75). Treatment for IBS-D focuses on regulating liver function and strengthening the spleen to improve gastrointestinal function, reduce depressive symptoms, enhance stress resilience, resolve dampness, and relieve abdominal pain (76). After diagnosis, symptom relief and quality of life improvement can be achieved by adjusting patients’ mindset and shifting attention away from symptom-related concerns.

Clinical data suggest that liver-regulating and intestine-moistening therapy reduces abdominal pain and improves constipation, potentially by lowering plasma levels of VIP and SS (74). Intestinal motility disorders are key pathophysiological mechanisms in IBS, with IBS-D patients showing accelerated colonic and small intestinal transit, and IBS-C patients experiencing delayed colonic transit (77). Kuan Zhong Jie Yu Tang, a classical TCM formula for liver depression-spleen deficiency, regulates the brain-gut axis by reducing VIP, SS, and 5-HT levels, alleviating visceral hypersensitivity and improving intestinal motility, thus enhancing IBS symptoms and quality of life (74).

Classical TCM formulas like TXYF, and XYS, are commonly used to treat IBS-D symptoms. XY-S, for liver-spleen disharmony, is often used for depression and functional dyspepsia. It contains *Bupleurum chinense* DC., *Angelica sinensis* (Oliv.) Diels, *Paeonia lactiflora* Pall. (Baishao), *Atractylodes macrocephala* Koidz., *Poria cocos* (Schw.) Wolf., *Glycyrrhiza glabra* L., *Mentha canadensis* L., *Zingiber officinale* Roscoe.

Notably, TXYF, a classic liver-spleen harmonizing prescription, has been modified for IBS-D by many herbal practitioners (78). It contains *Saposhnikovia divaricata* (Turcz. ex Ledeb.) Schischk., *Atractylodes macrocephala* Koidz., *Paeonia lactiflora* Pall., and *Citrus reticulata* Blanco. These herbs relieve IBS-D symptoms by harmonizing spleen and liver functions and have been used for centuries to alleviate abdominal pain with diarrhea.

Yuan et al. (79) demonstrated that TXYF exerts antidiarrheal effects by (i) inhibiting colonic hypermotility via blocking extracellular Ca^2+^ influx in rat colonic smooth muscle cells (CSMCs) and (ii) suppressing intestinal mucosal MC activation. TXYF also downregulates Protease-activated receptor-2 (PAR-2) and Proto-oncogene fos (c-Fos) expression, while reducing Tumor necrosis factor-*α* (TNF-α) and Interleukin-6 (IL-6) levels in post-inflammatory IBS rats (80). Additionally, TXYF lowers levels of gastrointestinal hormones, including 5-HT, Substance P (SP), VIP, SS, and cholecystokinin (CCK) in IBS rats and human patients (81).

TXYF has a long history in China for treating diarrhea. A systematic review with 1,125 participants showed TXYF was more effective than conventional drugs in treating IBS (risk ratio: 1.35; confidence interval: 1.21–1.50) (82). It alleviates post-infectious IBS symptoms by inhibiting PAR-2 expression, reducing SP and TNF-α levels, and decreasing IL-6 in colonic mucosa, alleviating hypersensitivity, diarrhea, and fecal serine protease activity (83). TXYF also improves intestinal barrier function by inhibiting NF-κB (Nuclear Factor Kappa-Light-Chain-Enhancer of Activated B Cells) and Notch signaling pathways (84). Meta-analysis (85) shows TXYF and its modifications exhibit significant efficacy and safety compared to conventional drugs or placebo. However, its exact therapeutic targets and molecular mechanisms remain unclear.

In a study, 60 IBS patients were randomly assigned to a control group (n = 20) receiving pinaverium bromide tablets, and a treatment group (n = 40) receiving TXYF, both in addition to conventional therapy. TXYF significantly improved IBS-D symptoms (86), potentially by modulating gut microbiota, alleviating visceral hypersensitivity, regulating 5-HT levels, and inhibiting colonic contractions (87).

**Figure 8:**
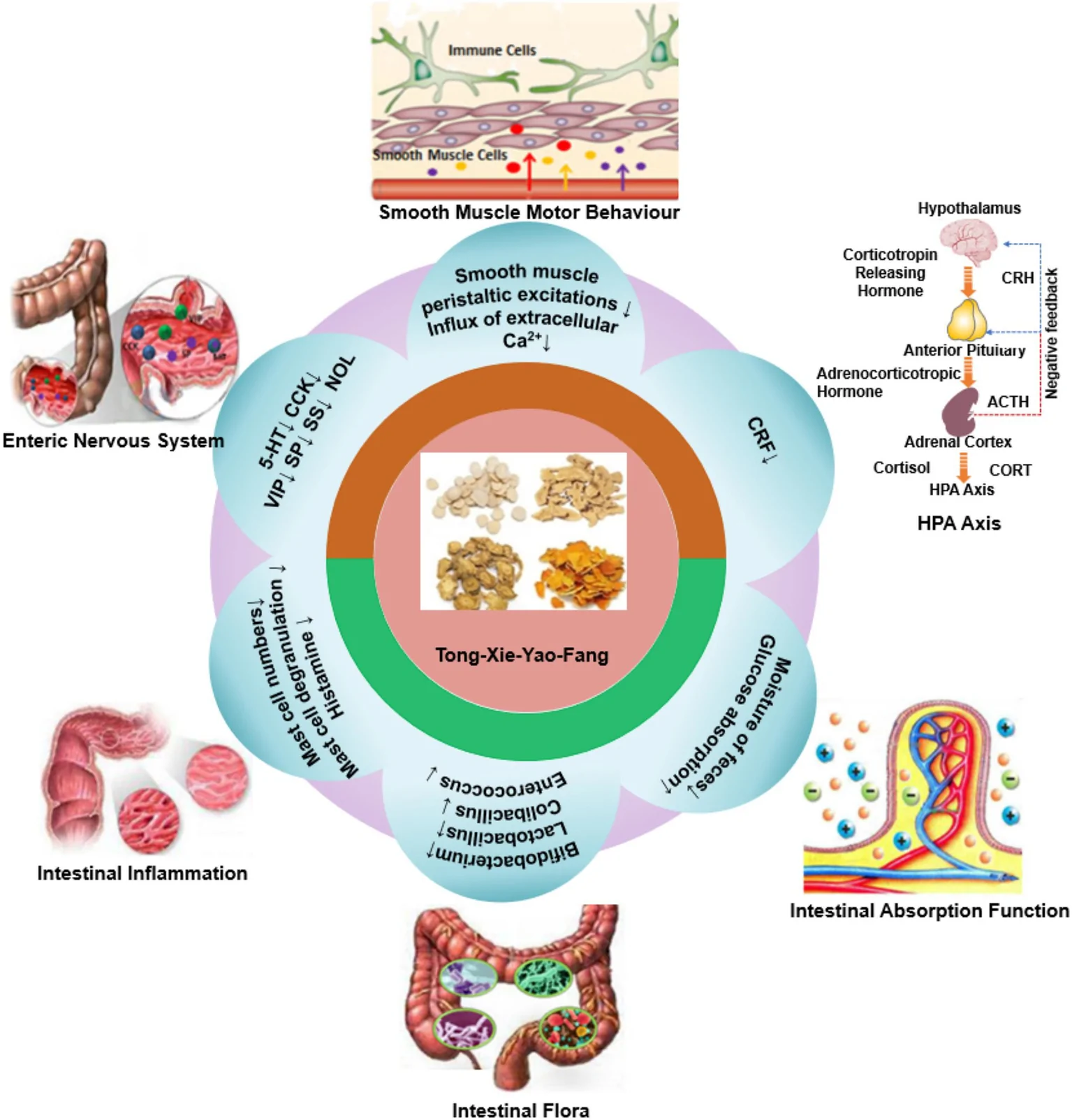
Multi-targeted treatment of pain and diarrhea remedies (115).

TXYF is a well-studied herbal formula for IBS, exemplifying the multi-target therapeutic potential of traditional herbal medicine, as shown in Figure 8. Given the complex pathogenesis of IBS, targeting a single pathway may be insufficient to address this multifactorial condition. Thus, there is an urgent need to develop novel multi-target therapies that can more effectively address both the core symptoms and comorbidities of IBS. Figure 8 illustrates a multi-target therapeutic mechanism of TXYF, integrating neurological, endocrine, and microbiological research. It includes the impact of intestinal inflammation on smooth muscle motility, modulation of gut flora by TXYF, involvement of the HPA axis in stress responses, and the influence of microbiota on intestinal absorption. TXYF modulates gut microbiota by increasing beneficial bacteria such as *Bifidobacterium* (B.) and *Lactobacillus* (L.), while reducing harmful species like *Escherichia* (Es.) *coli* and *Enterococcus* (Ec.). These findings highlight the multifactorial mechanisms underlying gut health, involving neural, immune, endocrine, and microbial interactions.

XYS enhances pain threshold and suppresses visceral hypersensitivity by downregulating 5-HT3 receptor expression, upregulating 5-HT4 receptor expression, and reducing serum cortisol levels (88). Additionally, XYS normalizes intestinal microbiota dysbiosis in patients with functional dyspepsia characterized by liver-spleen disharmony. This effect is associated with a decreased relative abundance of Firmicutes (Firm.), Proteobacteria (Prot.) and Cyanobacteria (Cyan.), and an increased abundance of Bacteroidetes (Bact.) (89).

Tongxie Antidiarrheal Decoction consists of seven herbs: *Atractylodes macrocephala* Koidz., *Paeonia lactiflora* Pall., *Coptis chinensis* Franch., *Prunus mume* (Siebold) Siebold & Zucc., *Citrus reticulata* Blanco, *Periostracum Cicadae*, and *Zingiber officinale* Roscoe. It alleviates IBS symptoms by reducing visceral hypersensitivity, inhibiting MC infiltration, and modulating 5-HT levels (85). However, the exact molecular targets and mechanisms remain unclear. Formulas based on *Atractylodes macrocephala* Koidz. (Baizhu) and *Paeonia lactiflora* Pall. are commonly used in TCM for treating diarrhea, bowel sounds, and spleen deficiency (90). *Atractylodes macrocephala* Koidz dries dampness, strengthens the spleen, and relieves diarrhea, while *Paeonia lactiflora* Pall purges fire, pacifies the liver, and relaxes spasms. Modern clinical practice has shown that compound formulas based on these herbs are effective in IBS management (91).

#### 5.1.3 Treating IBS from the spleen

In clinical practice, the spleen deficiency pattern is a prevalent TCM syndrome observed in patients with IBS-D. Shenling Baizhu Decoction, a classical TCM formula, is widely used to treat this syndrome and has been shown to alleviate spleen deficiency symptoms in IBS-D patients. However, its precise mechanism of action remains unclear. Studies have demonstrated that Shenling Baizhu Decoction exerts therapeutic effects in rat models of IBS-D with spleen deficiency (28). It is composed of *Panax ginseng* C.A.Mey., *Poria cocos* (Schw.) Wolf., *Atractylodes macrocephala* Koidz., *Glycyrrhiza uralensis* Fisch, *Dioscorea deltoidea* Wall. ex Griseb, *Lablab purpureus* (L.) Sweet, *Nelumbo nucifera* Gaertn., *Coix lacryma-jobi* L., *Wurfbainia villosa* (Lour.) Skornick. & A.D.Poulsen, *Platycodon grandiflorus* (Jacq.) A.DC., and *Ziziphus jujuba* Mill. The underlying mechanism may involve modulation of the intestinal microbiota and associated metabolic pathways, thereby alleviating diarrheal symptoms (28). Specifically, Shenling Baizhu Decoction may enhance intestinal barrier function by regulating the gut microbiota and metabolic pathways in IBS-D rats with spleen deficiency, thus contributing to its therapeutic efficacy.

According to TCM theory, spleen deficiency leads to reduced production of essential nutrients and blood, which may impair overall metabolic function. Shenling Baizhu Decoction exhibits anticholinergic effects, enhancing intestinal digestive absorption while suppressing motility, thereby preventing diarrhea and abdominal pain (92).

Modern pharmacological studies confirm that Shenling Baizhu Decoction, a classical formula for invigorating the spleen and eliminating dampness, alleviates metabolic disturbances in IBS-D (52). Its mechanism involves two main pathways: (i) inhibiting the abnormally activated Trp-serotonin metabolic axis, and (ii) enhancing the Trp-indole metabolic pathway. This dual-pathway regulation restores Trp metabolic balance in IBS-D model mice with spleen deficiency, exerting therapeutic effects. 5-HT, concentrated in neuronal synapses, acts as a neurotransmitter by binding to 5-HT1A receptors. It is primarily removed from the synaptic cleft through reuptake via the serotonin transporter (SERT), reducing synaptic 5-HT concentration and terminating its activity (93). Serum metabolites are processed through glycerophospholipid, Trp, and tyrosine metabolic pathways.

**Figure 9:**
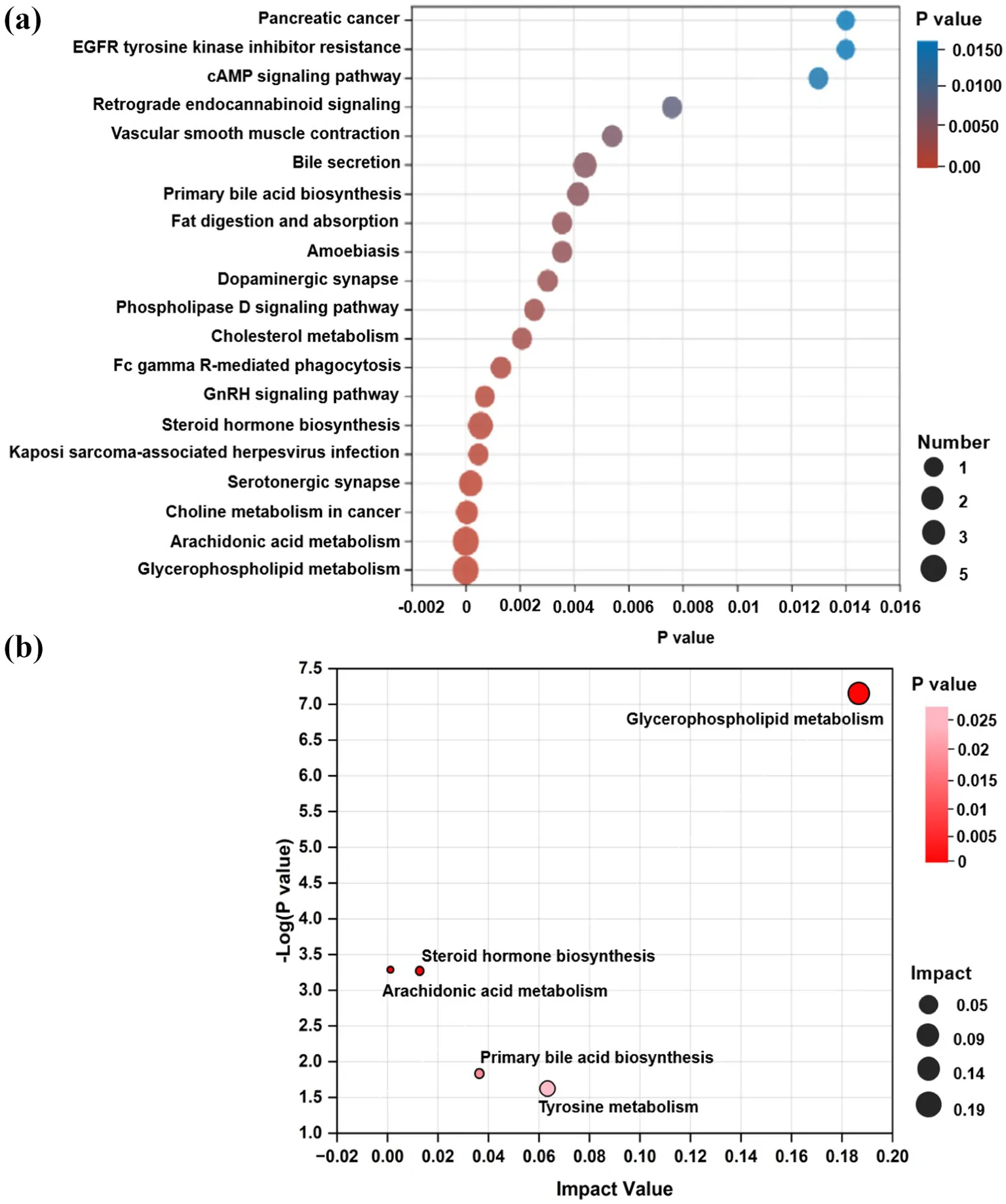
KEGG pathway analysis of differential metabolites in the *Shen Ling Bai Zhu Decoction* group and the IBS-D model group: **(a)** KEGG enrichment analysis of serum differential metabolites; **(b)** KEGG topology analysis of serum differential metabolites (28).

Furthermore, Tang et al. (28) conducted Kyoto Encyclopedia of Genes and Genomes (KEGG) pathways analysis of differential metabolites between the Shenling Baizhu Decoction and IBS-D model groups, as shown in Figure 9. Figure 9a reflects significant enrichment of multiple biological processes such as pancreatic cancer, Epidermal Growth Factor Receptor (EGFR) tyrosine kinase inhibitor resistance, and Cyclic Adenosine Monophosphate (cAMP) signaling pathway. Notably, the small *p*-value for EGFR tyrosine kinase inhibitor resistance suggests strong statistical significance. Figure 9b demonstrates the impact values of various pathways, including glycerophospholipid metabolism, steroid hormone biosynthesis, and aromatic amino acid metabolism. Larger impact value observed for glycerophospholipid metabolism and steroid hormone biosynthesis suggest substantial influence under experimental conditions.

In summary, Trp metabolism and steroid hormone biosynthesis are key regulatory pathways for intestinal metabolites, while glycerophospholipid metabolism and steroid hormone biosynthesis regulate serum metabolites. All of these pathways are modulated by Shenling Baizhu Decoction. Previous studies (38, 94) have also shown a close link between the serotonin synaptic signaling pathway and the pathogenesis of IBS.

Similarly, TCM has shown effectiveness in treating IBS-C. In an IBS-C rat model, the Mazhi Jiangzhuo Fang (a Chinese herbal formula for turbidity syndrome is shown in Table 3) is composed of *Sodium sulfate decahydrate*, *Citrus × aurantium* f*. aurantium*, *Magnolia officinalis* Rehder & E. H. Wilson, *Rheum palmatum* L., and *Ephedra sinica* Stapf. It alleviates symptoms associated with phlegm turbidity obstructing middle jiao pattern of IBS, potentially by enhancing small intestinal motility. Pharmacological studies have shown that *Citrus × aurantium* f*. aurantium* can promote small intestinal propulsion by increasing secretion of gastric actin, and can inhibit the expression of pro-inflammatory factors such as IL-6, thus improving the constipation symptoms of IBS-C (95). Relevant experimental studies have also shown that Mazhi Jiangzhuo Fang can increase the intestinal motility and improve the stool shapes of IBS-C rats (96).

**Table 3:** TCM interventions for IBS: patterns, mechanisms, and evidence.

Intervention	IBS pattern/indication	Key components (Latin names)	Proposed mechanisms/pharmacology	Evidence type	Major limitations	Representative references
Mazhi Jiangzhuo Fang	Phlegm-turbidity obstructing the middle jiao (IBS-C)	Sodium sulfate decahydrate, *Citrus × aurantium* f. *aurantium*, *Magnolia officinalis* Rehder & E.H.Wilson, *Rheum palmatum* L., *Ephedra sinica* Stapf	Enhances small intestinal motility, suppresses IL-6 expression, and attenuates gut inflammation	Animal studies (rats)	Limited clinical validation; mechanisms derived primarily from animal models	Huang et al. (5); Yang et al. (96)
Mazi Ren Pill (Tiaowei Maren Wan)	Constipation with stomach-heat and intestinal dryness (IBS-C)	*Cannabis sativa* L., *Citrus × aurantium* f. *aurantium*, *Paeonia lactiflora* Pall., *Prunus armeniaca* L., *Rheum palmatum* L., *Magnolia officinalis* Rehder & E.H.Wilson	Reduces visceral hypersensitivity, lowers serotonin levels, and modulates pro- and anti-inflammatory cytokines	Animal studies; clinical trials	Small sample sizes and heterogeneous patient populations	Lin et al. (97); Fu et al. (98)
Xiangsha Liujunzi Decoction	Spleen and stomach deficiency (IBS-D/C)	*Dolomiaea costus* (Falc.) Kasana & A.K.Pandey, *Wurfbainia villosa* (Lour.) Skornick. & A.D.Poulsen, *Panax ginseng* C.A.Mey., *Poria cocos* (Schw.) Wolf., *Atractylodes macrocephala* Koidz., *Glycyrrhiza glabra* L., *Citrus reticulata* Blanco, *Pinellia ternata* (Thunb.) Makino	Improves gastrointestinal motility, regulates intestinal microbiota, and alleviates intestinal inflammation and bacterial overgrowth	Clinical studies; experimental evidence	Limited multicenter randomized controlled trials and heterogeneous outcome measures	Shih et al. (99)
Fuzi Lizhong Pill	Spleen-kidney yang deficiency (IBS-D)	*Aconitum carmichaelii* Debeaux, *Panax ginseng* C.A.Mey., *Atractylodes macrocephala* Koidz., *Glycyrrhiza glabra* L., *Zingiber officinale* Roscoe	Modulates gut microbiota diversity and regulates immune and inflammatory responses	Animal studies	Lack of large-scale clinical trials and long-term follow-up data	Zhen et al. (100)
Sishen Pill	Chronic diarrhea associated with spleen-kidney yang deficiency (IBS-D)	*Cullen corylifolium* (L.) Medik., *Tetradium ruticarpum* (A.Juss.) T.G.Hartley, *Myristica fragrans* Houtt., *Schisandra chinensis* (Turcz.) Baill., *Ziziphus jujuba* Mill., *Zingiber officinale* Roscoe	Protects intestinal barrier integrity, restores microbial diversity, inhibits pathogenic bacteria, suppresses CRF-CREB signaling, and alleviates visceral hypersensitivity	Animal studies	Mechanistic evidence mainly derived from rat models, with limited clinical validation	Liu et al. (101); Wei et al. (53)
Shenling Baizhu Decoction	Spleen deficiency with dampness accumulation (IBS-D)	*Panax ginseng* C.A.Mey., *Atractylodes macrocephala* Koidz., *Poria cocos* (Schw.) Wolf., *Dolomiaea costus* (Falc.) Kasana & A.K.Pandey, *Citrus reticulata* Blanco, *Pinellia ternata* (Thunb.) Makino, *Glycyrrhiza glabra* L., etc.	Regulates Trp-serotonin metabolism, enhances the Trp-indole pathway, restores Trp homeostasis, exerts anti-inflammatory effects, and modulates the gut–brain axis	Animal studies	Translational relevance to humans remains uncertain, and formulations vary considerably among studies	Guzel et al. (52); Rudnick et al. (93)
Curcumin	IBS (general)	—	Modulates neurotransmitter systems and BDNF/CREB signaling pathways within the CNS and gut	Animal studies	Limited direct evidence in IBS patients; optimal dosing remains unclear	Yu et al. (102)
Acupuncture/moxibustion	IBS-D with various TCM patterns	—	Reduces CRF and SP levels; regulates gut-brain peptides, gastrointestinal motility, visceral hypersensitivity, immune responses, neurotransmitters, and the brain-gut-microbiota axis	Clinical studies	Small sample sizes, heterogeneous study designs, and variability in acupoint selection and treatment protocols	Tang et al. (110); Liao et al. (109); Yang et al. (108); Yang et al. (107)
Psychotherapy/mind–body therapies (CBT, MBT, MBSR, positive psychology)	IBS-D	—	Improves emotional regulation, reduces disease-related distress, and modulates brain-gut interactions	Clinical studies	Intervention protocols vary considerably, and long-term follow-up data are limited	Wu et al., (104); Jun et al. (105); Zhang et al. (106)

Level of evidence was categorized according to the overall strength of currently available evidence, including clinical practice guidelines, systematic reviews, randomized controlled trials, observational studies, and experimental research. The proposed mechanisms represent interpretations based on currently available evidence with varying levels of experimental and clinical support and should not be considered definitive mechanistic confirmation.

Mazi Ren Pill (a classical Chinese herbal formula for constipation) contains *Cannabis sativa* L., *Citrus × aurantium* f. *aurantium*, *Paeonia lactiflora* Pall., *Prunus armeniaca* L., *Rheum palmatum* L.,and *Magnolia officinalis* Rehder & E.H.Wilson. It reduces pain in IBS with stomach heat and intestinal dryness pattern by decreasing visceral hypersensitivity and lowering serotonin levels in rat models (97).

Tiaowei Maren Wan is a TCM derived from the Treatise on Cold Damage and is indicated for the pattern of intestinal dryness and fluid depletion. Clinical trials have shown that it improves abdominal symptoms and constipation in IBS-C patients. Its therapeutic effects are associated with modulation of proinflammatory and anti-inflammatory cytokines, including reduced IL-6 levels and increased IL-10 levels (98).

Xiangsha Liujunzi Decoction (a Chinese herbal formula for deficiency of spleen and stomach) contains *Dolomiaea costus* (Falc.) Kasana & A.K.Pandey, *Wurfbainia villosa* (Lour.) Skornick. & A.D.Poulsen, *Panax ginseng* C.A.Mey., *Poria cocos* (Schw.) Wolf., *Atractylodes macrocephala* Koidz., *Glycyrrhiza glabra* L., C*itrus reticulata* Blanco, and *Pinellia ternata* (Thunb.) Makino. It has been shown to significantly relieve diarrheal symptoms in IBS patients by alternating gastrointestinal motility and fecal microflora, and intestinal inflammation or bacterial overgrowth (99).

The Fuzi Lizhong Pill is a classical Chinese herbal formula. It is used to treat spleen-kidney yang deficiency. This condition is marked by impaired digestive function, cold intolerance, and fatigue due to insufficient metabolic activity. The pill is composed of *Aconitum carmichaelii* Debeaux, *Panax ginseng* C.A.Mey., *Atractylodes macrocephala* Koidz., *Glycyrrhiza glabra* L.,and *Zingiber officinale* Roscoe. It modulates gut microbiota diversity and regulates inflammatory and immune responses. It is indicated for treating spleen-kidney yang deficiency syndrome in patients with IBS-D (100).

In patients with IBS-D, studies have shown that Sishen Pill (101) (a traditional Chinese herbal formula for treating chronic diarrhea and spleen-kidney yang deficiency) protects intestinal mucosal function by reducing the levels of Prot. and *Mycoplasma*, and increasing the abundance of *Clostridium*, *Bacillus*, and Firm. Sishen Pill is thought to inhibit pathogenic bacteria by competing for nutrients and maintaining metabolic balance. It consists of *Cullen corylifolium* (L.) Medik., *Tetradium ruticarpum* (A.Juss.) T.G.Hartley, *Myristica fragrans* Houtt., *Schisandra chinensis* (Turcz.) Baill., *Ziziphus jujuba* Mill, and *Zingiber officinale* Roscoe. It also helps restore gut microbiota diversity, increases microbial abundance and richness, and regulates intestinal microecological balance, thereby protecting the intestinal barrier and alleviating symptoms of IBS. Study has shown that the alcohol extract of *Cullen corylifolium* (L.) Medik.-*Myristica fragrans* Houtt. significantly decreases the expression of corticotropin-releasing factor (CRF), its receptor CRF-R1, protein kinase A (PKA), cAMP response element-binding protein (CREB), phosphorylated CREB (p-CREB), and the c-Fos in colonic tissues, thereby alleviating visceral hypersensitivity in rats. This effect may be mediated by modulation of the CRF-CREB signaling pathway, which plays a role in reducing intestinal hypersensitivity (53). Curcumin alleviates symptoms of IBS by modulating neurotransmitter levels, brain-derived neurotrophic factor (BDNF), and CREB signaling pathways in both the CNS and the peripheral gut (102).

In TCM theory, the spleen is regarded as responsible for transportation and transformation. Within this framework, spleen deficiency is considered an important pathological pattern in IBS and is often associated with dampness accumulation, weakness of the spleen and stomach, and obstruction of the middle jiao by phlegm and turbidity. Accordingly, therapeutic strategies typically focus on strengthening spleen function, eliminating dampness, resolving phlegm, and restoring gastrointestinal balance.

Experimental and clinical studies have suggested that TCM interventions targeting spleen-related dysfunction may influence several biological processes relevant to IBS, including gut microbiota composition, inflammatory responses, and brain-gut axis signaling. These mechanisms may contribute to improvements in gastrointestinal symptoms; however, the underlying biological pathways remain incompletely understood. Furthermore, the therapeutic efficacy of TCM for IBS-D associated with spleen deficiency, as well as its effects on gut microbiota and metabolic profiles, requires further confirmation through large-scale clinical studies with longer follow-up periods.

### 5.2 Emotional regulation

**Figure 10:**
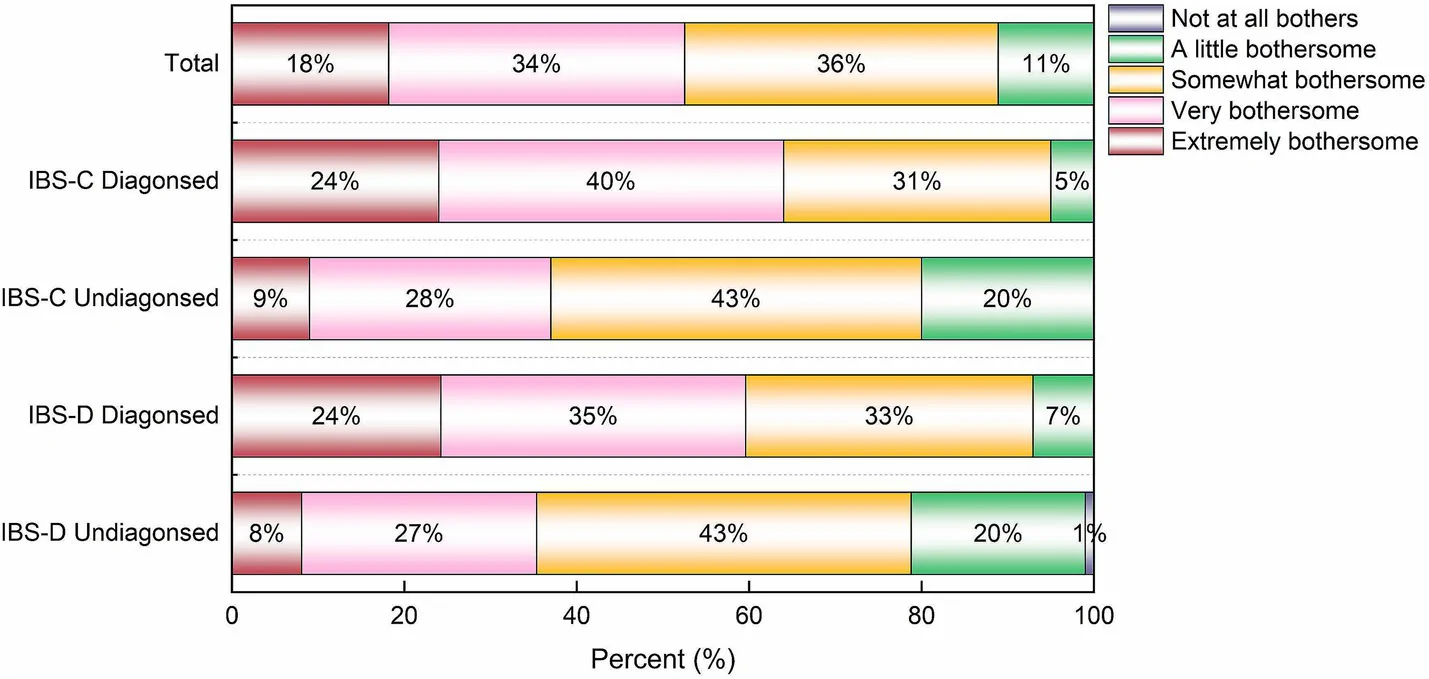
Research data on IBS symptoms and different levels of affective activity (reference website: https://www.multivu.com).

Figure 10 shows the data from a study commissioned by the American Gastroenterological Association (AGA) (103) investigating the correlation between IBS symptoms and affective states. The graph depicts the distribution of symptom-related distress across five severity levels: not at all bothers, a little bothersome, somewhat bothersome, very bothersome, and extremely bothersome. The data reveals that 34% of patients reported symptoms as very bothersome and 18% as extremely bothersome, totaling 52%. This implies that IBS has a substantial impact on daily activities of the patients. Moreover, among patients with IBS-C and IBS-D, those with a confirmed diagnosis reported higher levels of emotional distress (very bothersome and extremely bothersome) than those without a diagnosis. This indicates a clear association between disease recognition and emotional disturbance. Therefore, targeting IBS symptoms through emotion regulation from the TCM perspective represents a viable therapeutic approach.

In TCM, the liver is involved in emotional regulation. Liver stagnation may impair gastrointestinal function and contribute to neuroendocrine and immune dysregulation. These disruptions may ultimately impair brain function. Wu et al. (104) conclude that psychotherapy plays a significant role in the management of IBS-D. Liver-Spleen Oriented Therapy in IBS demonstrates significant clinical efficacy, with improvements in patient symptoms and quality of life. A variety of non-pharmacological therapies have also shown promise in the management of IBS. These include cognitive behavioral therapy (CBT), gut-directed hypnotherapy, mind–body therapy (MBT), positive psychology interventions such as mindfulness-based stress reduction (MBSR), herbal remedies, and probiotic therapy (105, 106).

### 5.3 Other external Chinese medicine treatments

With limited conventional IBS treatments, increasing numbers of patients turn to complementary and alternative medicine (CAM). TCM therapies, particularly acupuncture- and moxibustion-based interventions, have shown potential benefits in improving IBS-D symptoms and are generally associated with relatively few adverse effects in clinical studies (107, 108).

**Figure 11:**
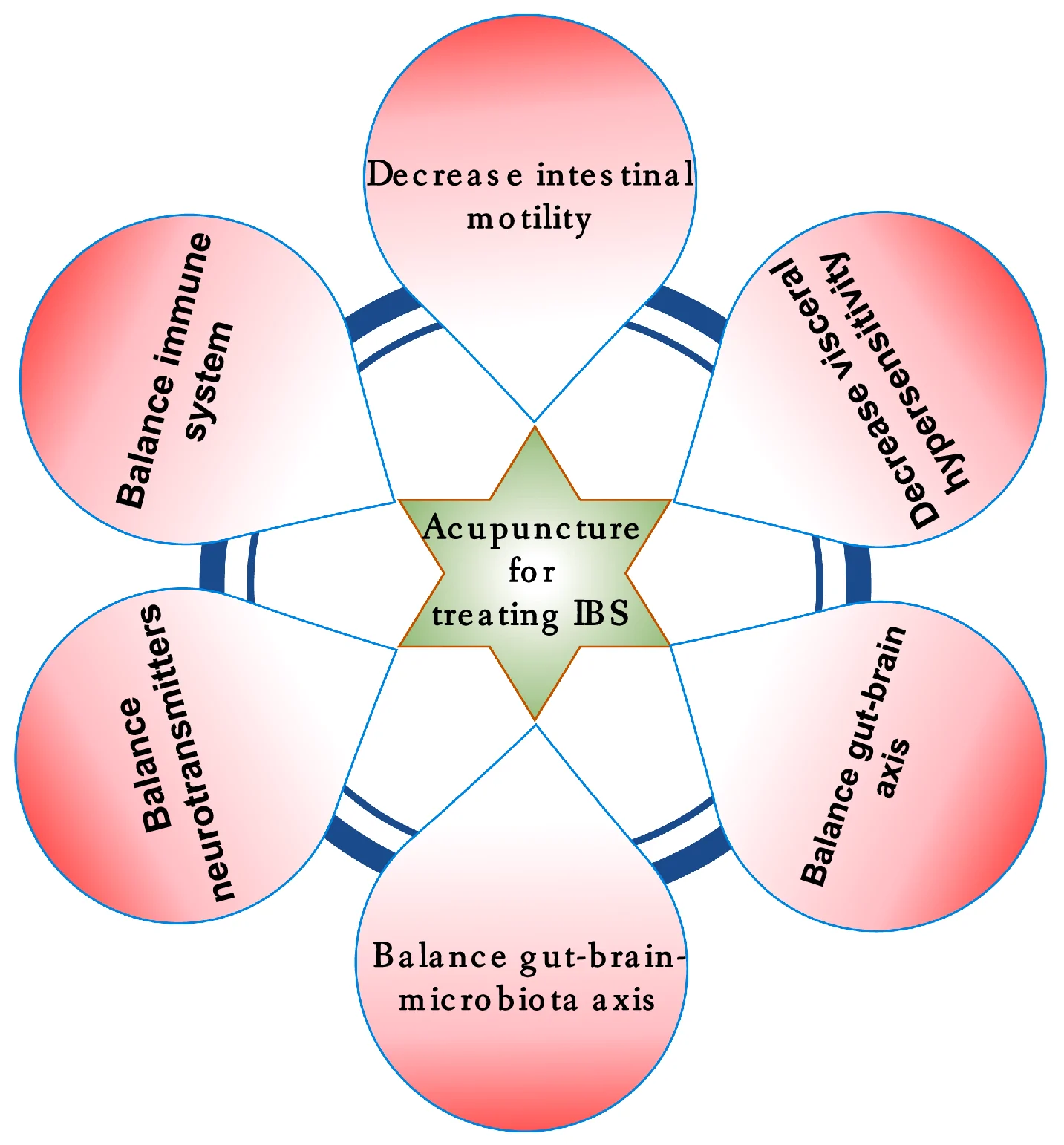
Multidimensional approach to IBS: modulation of gut function, immune system and gastrointestinal axis balance.

Moxibustion at Stomach Meridian, point 25 (ST25) and Stomach Meridian, point 37 (ST37) reduces CRF and SP, easing visceral hypersensitivity (109). Acupuncture at Stomach Meridian, point 25 (ST36), ST37, and Liver Meridian, point 3 (LR3) regulates gut-brain peptides and motility (110). As shown in Figure 11, it modulates motility, hypersensitivity, immunity, neurotransmitters, and the brain-gut-microbiota axis, supporting integrative therapy.

## 6 Effectiveness of TCM clinical interventions

Evidence-based studies have reported potential benefits of certain TCM interventions for IBS. However, much of the available clinical evidence is derived from single-center studies with relatively small sample sizes, and high-quality multicenter randomized controlled trials and recent meta-analyses remain limited (67, 111).

From a clinical perspective, TCM is characterized by individualized treatment based on syndrome differentiation and holistic regulation. Some studies have reported improvements in abdominal pain, bowel habits, and patient-reported outcomes following TCM interventions. Nevertheless, heterogeneity in diagnostic criteria, treatment protocols, and outcome measures complicates comparisons across studies and limits the strength of current conclusions. Several potential mechanisms of TCM interventions in IBS have been proposed, but most evidence remains preclinical and requires further clinical validation (53).

Within the theoretical framework of TCM, IBS is commonly interpreted as involving dysfunction of the spleen, liver, and, in some patients, the heart. Syndrome differentiation provides a conceptual basis for individualized treatment strategies. While this framework may help guide clinical practice, its biological correlates remain incompletely understood and warrant further investigation using modern biomedical approaches.

Overall, TCM offers a potentially valuable complementary approach for IBS management. However, further standardization of syndrome classification, treatment protocols, and efficacy assessment is needed to strengthen the evidence base and facilitate international acceptance.

Across different TCM interventions, several recurring biological pathways have been identified, including regulation of the brain–gut axis, modulation of gut microbiota composition, immune and inflammatory responses, Trp metabolism, and visceral sensitivity. Notably, many of these pathways correspond to functional domains traditionally attributed to the heart, liver, and spleen in TCM theory. Such convergence may provide a mechanistic basis for interpreting classical concepts within a modern biomedical framework. However, current evidence is derived predominantly from preclinical studies, and further validation using quantitative biomarkers and clinical endpoints is required (53, 54). Rather than serving as an alternative to conventional medicine, TCM may be viewed as a complementary approach that could be integrated with evidence-based Western therapies according to individual patient characteristics and treatment goals.

## 7 Limitations and future perspectives

Current evidence on TCM for IBS has several limitations. Methodological quality varies considerably across studies, with common issues including small sample sizes, inadequate blinding, and limited long-term follow-up. Most studies primarily focus on short-term symptom improvement, whereas data on long-term recurrence rates, sustained treatment responses, and quality-of-life outcomes remain insufficient. Furthermore, high-quality multicenter randomized controlled trials and recent large-scale evidence syntheses remain relatively scarce. Heterogeneity in syndrome differentiation, herbal formulas, treatment duration, and outcome measures further limits comparability and generalizability. Potential biases (selection, reporting, publication) and strong placebo responses-particularly relevant in patient-reported outcomes-may overestimate treatment effects.

Moreover, although mechanistic studies have proposed pathways involving the brain-gut axis, gut microbiota, immune regulation, and visceral hypersensitivity, many findings remain preliminary. Future studies should adopt standardized diagnostic criteria, rigorous randomized controlled designs, larger sample sizes, and internationally recognized outcome measures. In addition, although mind–body integrated therapy has shown potential benefits in IBS management, standardized clinical implementation protocols, patient selection criteria, and treatment algorithms remain insufficiently defined. The clinical translation of the heart-liver-spleen framework remains limited by the lack of standardized biomarkers, assessment tools, and intervention protocols. Objective criteria for identifying heart-liver-spleen imbalance remain lacking, and future studies should evaluate biomarker- and phenotype-based stratification strategies to support precision medicine in IBS. Mechanistic research should prioritize the identification and validation of biomarkers linked to TCM syndrome patterns, including neuroendocrine mediators, inflammatory cytokines, intestinal barrier markers, microbiome profiles, and metabolomic signatures. Particular attention should be given to the identification of objective biomarkers that can be linked to TCM syndrome patterns. Integrating multi-omics technologies, microbiome analyses, metabolomics, and quantitative clinical phenotyping may help establish more robust biological correlates for TCM diagnostic frameworks.

In addition to efficacy, future studies should place greater emphasis on safety outcomes. Potential concerns include adverse effects associated with long-term use of certain herbal medicines, herb-drug interactions, variability in herbal product quality, and contamination with heavy metals or other impurities. Standardized safety monitoring and long-term pharmacovigilance data remain limited. The real-world implementation of integrated TCM-Western medicine models may be limited by resource, training, and healthcare system factors, which warrant further investigation.

## 8 Conclusion

IBS is a multifactorial disorder characterized by complex interactions among the gastrointestinal tract, nervous system, immune responses, endocrine regulation, and gut microbiota. Within the theoretical framework of TCM, these manifestations may be interpreted through functional interactions among the heart, liver, and spleen, providing a holistic perspective for individualized diagnosis and treatment.

TCM-based approaches may contribute to IBS management through multiple complementary mechanisms, including modulation of gastrointestinal function, immune responses, and the brain–gut–microbiota axis. However, the proposed correspondence between the heart–liver–spleen framework and contemporary biomedical mechanisms should be regarded as a conceptual and hypothesis-generating model rather than a direct biological equivalence. The precise mechanistic relationships remain incompletely understood and require further validation through rigorous translational and clinical studies.

Despite increasing interest in TCM-based interventions for IBS, challenges remain regarding syndrome classification, treatment standardization, and evidence quality. Future studies integrating systems biology, multi-omics approaches, and well-designed clinical trials may further clarify the biological basis of TCM interventions and promote evidence-informed integration with contemporary biomedical research.

## Glossary

IBS: Irritable bowel syndrome
TCM: Traditional Chinese Medicine
IBS-D: Diarrhea-predominant IBS
IBS-C: Constipation-predominant IBS
IBS-M: Mixed IBS
IBS-U: Unclassified IBS
GERD: Gastroesophageal reflux disease
CNS: Central nervous system
SCFAs: Short-chain fatty acids
miRNAs: MicroRNAs
HPA: Hypothalamic–pituitary–adrenal
EECs: Enteroendocrine cells
IECs: Intestinal epithelial cells
PC: Paneth cell
IL-6: Interleukin-6
GABA: *γ*-Aminobutyric acid
5-HT(5-hydroxytryptamine): Serotonin
AMP: Antimicrobial peptide
HRV: Heart rate variability
ANS: Autonomic nervous system
LF: Low-frequency
HF: High-frequency
ENS: Enteric nervous system
DA: Dopamine
NA: Norepinephrine
Ach: Acetylcholine
CRH: Corticotropin-releasing hormone
ACTH: Adrenocorticotropic hormone
ECC: Enteric chromaffin cells
Trp: Tryptophan
FEED: Fiber, elevation, exercise, dietary modification
ACG: American College of Gastroenterology
LFD: Low FODMAP diet
FODMAPs: Fermentable oligosaccharides, disaccharides, monosaccharides, and polyols
FMT: Fecal microbiota transplantation
CDI: *Clostridioides* difficile infection
VIP: Vasoactive intestinal peptide
SS: Somatostatin
TXYF: Tong Xie Yao Fang
TWBXD: Tianwang Buxin Dan
XYS: Xiao Yao San
PAR-2: Protease-activated receptor-2
c-Fos: Proto-oncogene fos
TNF-*α*: Tumor necrosis factor-α
NF-κB: Nuclear factor kappa-light-chain-enhancer of activated B cells
SP: Substance P
SERT: Serotonin transporter
KEGG: Kyoto Encyclopedia of Genes and Genomes
EGFR: Epidermal growth factor receptor
cAMP: Cyclic adenosine monophosphate
CRF: Corticotropin-releasing factor
PKA: Protein kinase A
CREB: cAMP response element-binding protein
p-CREB: phosphorylated CREB
BDNF: Brain-derived neurotrophic factor
AGA: American Gastroenterological Association
CBT: Cognitive behavioral therapy
MBT: Mind–body therapy
MBSR: Mindfulness-based stress reduction
MC: Mast cell
CSMCs: Colonic smooth muscle cells
B.: *Bifidobacterium*
L.: *Lactobacillus*
Es.: *Escherichia*
Ec.: *Enterococcus*
Firm.: Firmicutes
Prot.: Proteobacteria
Cyan.: Cyanobacteria
Bact.: Bacteroidetes
CAM: Complementary and alternative medicine
