# Supplementary material for: Pathogenesis and therapeutic strategies for irritable bowel syndrome: an integrated perspective from dynamic balance theory of Chinese medicine
Source: Front Med (Lausanne). 2026 Aug 19;13:1823683. doi: 10.3389/fmed.2026.1823683 (PMC13537036; doi:10.3389/fmed.2026.1823683)
Supplement: Supplementary file 1 [file Supplementary_file_1.docx]

**Table S1 The effects of TCM formulas on IBS.**

| Formula | Composition | Action mechanism | References |
| --- | --- | --- | --- |
| Shenling Baizhu Decoction | Renshen (*Panax ginseng* C.A.Mey*.*),  Fuling (*Poria cocos* (Schw.) Wolf*.*),  Baizhu (*Atractylodes macrocephala* Koidz.),  Gancao (*Glycyrrhiza uralensis* Fisch)*,*  Shanyao *(Dioscorea deltoidea* Wall. ex Griseb.),  Baibiandou (*Lablab purpureus* subsp*. Purpureus*),  Lianzi (*Nelumbo nucifera* Gaertn*.*),  Yiyiren (*Coix lacryma-jobi* L.),  Sharen (*Wurfbainia villosa* (Lour.) skornick. & A.D.Poulsen),  Jiegeng (*Platycodon grandiflorus* (Jacq.) A.DC.),  Dazao (*Ziziphus jujuba* Mill*.*). | Inhibited the abnormally activated tryptophan-serotonin metabolic axis and enhanced the tryptophan-indole metabolic pathway, KEGG pathways. | Guzel T et al. (2022)  Rudnick G et al. (2019)  Tang B B et al. (2024) |
| Mazhi Jiangzhuo Fang | Mahuang (*Ephedra sinica* Stapf),  Zhishi (*Citrus × aurantium* f*. aurantium*),  Houpo (*Magnolia officinalis* Rehder & E.H.Wilson)*,*  Dahuang (*Rheum palmatum* L.),  Mangxiao (*Sodium sulfate decahydrate*). | Increased the intestinal motility and improve the stool shapes. | Yang Qian et al. (2015) |
| Mazi Ren Pill | Huomaren (*Cannabis sativa* L.),  Zhishi (*Citrus × aurantium* f. *aurantium*),  Baishao (*Paeonia lactiflora* Pall.),  Kuxingren (*Prunus armeniaca* L.),  Dahuang (*Rheum palmatum* L.),  Houpo (*Magnolia officinalis* Rehder & E.H.Wilson). | Decreased visceral hypersensitivity and lowered serotonin levels in rat models. | Lin Z Y et al. (2014) |
| Xiangsha Liujunzi Decoction | Muxiang (*Dolomiaea costus* (Falc.) Kasana & A.K.Pandey),  Sharen (*Wurfbainia villosa* (Lour.) Skornick. & A.D.Poulsen), Renshen (*Panax ginseng* C.A.Mey.),  Fuling (*Poria cocos* (Schw.) Wolf.),  Baizhu (*Atractylodes macrocephala* Koidz.),  Zhigancao (*Glycyrrhiza glabra* L.),  Chenpi (*Citrus reticulata* Blanco),  Banxia (*Pinellia ternata* (Thunb.) Makino). | Alternated gastrointestinal motility and fecal microflora, and intestinal inflammation or bacterial overgrowth. | Shih Y S et al. (2019) |
| Fuzi Lizhong Pill | Fuzi (Aconitum carmichaelii Debeaux),  Renshen (*Panax ginseng* C.A.Mey.),  Ganjiang (*Zingiber officinale* Roscoe),  Baizhu (*Atractylodes macrocephala* Koidz.),  Zhigancao (*Glycyrrhiza glabra* L.). | Modulated gut microbiota diversity and regulated inflammatory and immune responses. | Zhen Z et al. (2021) |
| Sishen Pill | Buguzhi (*Cullen corylifolium* (L.) Medik.),  Wuzhuyu (*Tetradium ruticarpum* (A.Juss.) T.G.Hartley),  Roudoukou (*Myristica fragrans* Houtt.),  Wuweizi (*Schisandra chinensis* (Turcz.) Baill.),  Shengjiang (*Zingiber officinale* Roscoe),  Dazao (*Ziziphus jujuba* Mill.) | Protected intestinal mucosal function, inhibited pathogenic bacteria, protected the intestinal barrier, modulated of the CRF-CREB signaling pathway. | Lin et al. (2014)  Mishima Y et al. (2021) |
| Tian Wang Bu Xin Dan | Dihuang (*Rehmannia glutinosa* (Gaertn.) Libosch. ex DC.)  Danggui (*Angelica sinensis* (Oliv.) Diels),  Maidong (*Ophiopogon japonicus* (Thunb.) Ker Gawl.),  Tiandong (*Asparagus cochinchinensis* (Lour.) Merr.),  Suanzaoren (*Ziziphus spinosa* Lam),  Baiziren (*Platycladus orientalis* (L.) Franco),  Yuanzhi (*Polygala tenuifolia* Willd.),  Fuling (*Poria cocos* (Schw.) Wolf.),  Wuweizi(*Schisandra chinensis* (Turcz.) Baill.),  Jiegeng (*Platycodon grandiflorus* (Jacq.) A.DC.),  Xuanshen (*Scrophularia ningpoensis* Hemsl.),  Renshen (*Panax ginseng* C.A.Mey.),  Danshen (*Salvia miltiorrhiza* Bunge),  Zhusha (Cinnabar). | Regulated the intestinal flora and 5-HT level. | Yang et al. (2025) |
| Kuan Zhong Jie Yu Tang | Renshen (*Panax ginseng* C.A.Mey.),  Baizhu (*Atractylodes macrocephala* Koidz.),  Fuling (*Poria cocos* (Schw.) Wolf*.*),  Danggui (*Angelica sinensis* (Oliv.) Diels),  Baishao (*Paeonia lactiflora* Pall.),  Zhike (*Citrus × aurantium* f. *aurantium*),  Sharen (*Wurfbainia villosa* (Lour.) Skornick. & A.D.Poulsen),  Zhizi (*Plumeria rubra* L. Gardenia jasminoides J.Ellis),  Bohe (*Mentha spicata* L.). | Regulated the brain-gut axis further by reducing plasma levels of VIP, SS and 5-HT. | Xu YY et al. (2014) |
| Tong Xie Yao Fang | Chenpi (*Citrus reticulata* Blanco),  Baizhu (*Atractylodes macrocephala* Koidz.),  Baishao (*Paeonia lactiflora* Pall.),  Fangfeng (*Saposhnikovia divaricata (Turcz. ex Ledeb.) Schischk.*). | Blocked extracellular Ca²⁺ influx, suppressed intestinal mucosal mast cell activation, downregulated protease-activated receptor-2 (PAR-2) and proto-oncogene fos (c-Fos) expression while reducing tumor necrosis factor-α (TNF-α) and interleukin-6 (IL-6) levels,reduce levels of 5-HT, SP, VIP, SS, and CCK.  NF-κB and Notch signaling pathways. | Yuan J Y et al. (2009)  Ma X et al. (2017)  Rui Feng L et al. (2018)  Bian Z et al. (2006)  Hu X et al. (2013)  Hou Q et al. (2019) |
| Xiaoyao San | Chaihu (*Bupleurum chinense* DC.),  Danggui (*Angelica sinensis* (Oliv.) Diels),  Baishao (*Paeonia lactiflora* Pall.),  Baizhu (*Atractylodes macrocephala* Koidz.),  Fuling (*Poria cocos* (Schw.) Wolf.),  Zhigancao (*Glycyrrhiza glabra* L.),  Bohe (*Mentha* *canadensis* L.),  Shengjiang (*Zingiber officinale* Roscoe). | Enhanced pain threshold and suppressed visceral hypersensitivity by downregulating 5-HT3 receptor expression, upregulating 5-HT4 receptor expression, and reducing serum cortisol levels. Decreased relative abundance of *Firmicutes*, *Proteobacteria* and *Cyanobacteria*, and an increased abundance of *Bacteroidetes.* | Qiu J J et al. (2017)  Tan X et al. (2020) |
| Ban Xia Xie Xin Tang | Banxia (*Pinellia ternata* (Thunb.) Makino),  Huangqin (*Scutellaria baicalensis* Georgi),  Huanglian (Coptis chinensis Franch.),  Ganjiang (*Zingiber officinale* Roscoe),  Renshen (*Panax ginseng* C.A.Mey.),  Zhigancao (*Glycyrrhiza glabra* L.),  Dazao (Ziziphus jujuba Mill.). | Reduced TNF-α and IL-6 secretion. It relieved gastrointestinal pain, enhanced motility and emptying, protected the intestinal mucosa, and reduced visceral sensitivity. | Zou Defang et al. (2024) |
| Tongxie Antidiarrheal Decoction | Baizhu (*Atractylodes macrocephala* Koidz.),  Baishao (*Paeonia lactiflora* Pall.),  Huanglian (*Coptis chinensis* Franch.),  Paojiang (*Zingiber officinale* Roscoe),  Chenpi (*Citrus reticulata* Blanco).,  Chantui (*Periostracum Cicadae*),  Wumei (*Prunus mume* (Siebold) Siebold & Zucc.). | Reduced visceral hypersensitivity, inhibited mast cell infiltration, and modulated 5-HT levels. | Zhou Y et al. (2019) |

**Table S2 The Source species of TCM involved in this work.**

| No. | Chinese Name | Family Name | Latin Name | Source species |
| --- | --- | --- | --- | --- |
| 1 | Baiziren | Cupressaceae | *Platycladi Semen* | *Platycladus orientalis* (L.) Franco |
| 2 | Suanzaoren | Rhamnaceae | *Ziziphi Spinosae Semen* | *Ziziphus spinosa* Lam |
| 3 | Maidong | Liliaceae | *Ophiopogonis Radix* | *Ophiopogon japonicus* (Thunb.) Ker Gawl. |
| 4 | Xuanshen | Scrophulariaceae | *Scrophulariae Radix* | *Scrophularia ningpoensis* Hemsl. |
| 5 | Banxia | Araceae | *Pinelliae Rhizoma* | *Pinellia ternata* (Thunb.) Makino |
| 6 | Huangqin | Labiatae | *Scutellariae Radix* | *Scutellaria baicalensis* Georgi |
| 7 | Huanglian | Ranunculaceae | *Coptidis Rhizoma* | Coptis chinensis Franch. |
| 8 | Ganjiang | Zingiberaceae | *Zingiberis Rhizoma* | *Zingiber officinale* Roscoe |
| 9 | Renshen | Araliaceae | *Ginseng Radix Et Rhizoma* | *Panax ginseng* C.A.Mey. |
| 10 | Gancao | Leguminosae | *Glycyrrhizae Radix Et Rhizoma* | *Glycyrrhiza uralensis* Fisch  *Glycyrrhiza inflata* Batalin  *Glycyrrhiza glabra* L. |
| 11 | Baizhu | Compositae | *Atractylodis Macrocephalae Rhizoma* | *Atractylodes macrocephala* Koidz. |
| 12 | Baishao | Ranunculaceae | *Paeoniae Radix Alba* | *Paeonia lactiflora* Pall. |
| 13 | Fangfeng | Umbelliferae | *Saposhnikoviae Radix* | *Saposhnikovia divaricata* (Turcz. ex Ledeb.) Schischk. |
| 14 | Chenpi | Rutaceae | *Citiri Reticulatae Pericarpium* | *Citrus reticulata* Blanco |
| 15 | Chaihu | Umbelliferae | *Bupleuri Radix* | *Bupleurum chinense* DC.  *Bupleurum scorzonerifolium* Willd. |
| 16 | Danggui | Umbelliferae | *Angelicae Sinensis Radix* | *Angelica sinensis* (Oliv.) Diels |
| 17 | Fuling | Polyporaceae | *Poria* | *Poria cocos* (Schw.) Wolf. |
| 18 | Zhigancao | Leguminosae | *Glycyrrhizae Radix Et Rhizoma Praeparata Cum Melle* | *Glycyrrhiza glabra* L. |
| 19 | Bohe | Labiatae | *Menthae Haplocalycis Herba* | *Mentha spicata* L. |
| 20 | Shengjiang | Zingiberaceae | *Zingiberis Rhizoma Recens* | *Zingiber officinale* Roscoe |
| 21 | Paojiang | Zingiberaceae | *Rhizoma Zingiberis Praeparatum* | Zingiber officinale Roscoe |
| 22 | Chantui | Cicadidae | *Cicadae Periostracum* | *Salvia miltiorrhiza* Bunge |
| 23 | Wumei | Rosaceae | *Mume Fructus* | *Prunus mume* (Siebold) Siebold & Zucc*.* |
| 24 | Shanyao | Dioscoreaceae | *Dioscoreae Rhizoma* | *Dioscorea deltoidea* Wall. ex Griseb. |
| 25 | Baibiandou | Leguminosae | *Lablab Semen Album* | *Lablab purpureus* subsp. *Purpureus* |
| 26 | Lianzi | Nymphaeaceae | *Nelumbinis Semen* | *Nelumbo nucifera* Gaertn. |
| 27 | Yiyiren | Coicis Semen | *Gramineae* | *Coix lacryma-jobi* L. |
| 28 | Sharen | Zingiberaceae | *Amomi Fructus* | *Wurfbainia villosa* (Lour.) skornick. & A.D.Poulsen |
| 29 | Jiegeng | Campanulaceae | *Platycodonis Radix* | *Platycodon grandiflorus* (Jacq.) A.DC. |
| 30 | Dazao | Rhamnaceae | *Jujubae Fructus* | *Ziziphus jujuba* Mill. |
| 31 | Mahuang | Ephedraceae | *Ephedrae Herba* | *Ephedra sinica* Stapf  *Ephedra intermedia* Schrenk & C.A.Mey.  *Ephedra equisetina* Bunge |
| 32 | Zhishi | Rutaceae | *Aurantii Fructus Immaturus* | *Citrus* × *aurantium* f. *aurantium* |
| 33 | Houpo | Magnoliaceae | *Magnoliae Officinalis Cortex* | *Magnolia officinalis* Rehder & E.H.Wilson  *Magnolia officinalis var. biloba* Rehder & E.H.Wilson |
| 34 | Dahuang | Polygonaceae | *Rhei Radix Et Rhizome* | *Rheum palmatum* L.  *Rheum officinale* Baill.  *Rumex acetosa* L. |
| 35 | Mangxiao | — | *Natrii Sulfas* | *Mirabilite* |
| 36 | Huomaren | Moraceae | *Cannabis Fructus* | *Cannabis sativa* L. |
| 37 | Kuxingren | Rosaceae | *Armeniacae Semen Amarum* | *Prunus armeniaca* L. |
| 38 | Muxiang | Compositae | *Aucklandiae Radix* | *Dolomiaea costus* (Falc.) Kasana & A.K.Pandey |
| 39 | Fuzi | Ranunculaceae | *Aconiti Lateralis Radix Praeparata* | *Cyperus rotundus* L. |
| 40 | Buguzhi | Leguminosae | *Psoraleae Fructus* | *Cullen corylifolium* (L.) Medik. |
| 41 | Wuzhuyu | Rutaceae | *Euodiae Fructus* | *Tetradium ruticarpum* (A.Juss.) T.G.Hartley |
| 42 | Roudoukou | Myristicaceae | *Myristicae Semen* | *Myristica fragrans* Houtt. |
| 43 | Wuweizi | Magnoliaceae | *Schisandrae Chinensis Fructus* | *Schisandra chinensis* (Turcz.) Baill.  *Schisandra arisanensis* Hayata  *Schisandra bicolor* W.C.Cheng |
| 44 | Huangqi | Leguminosae | *Astragali Radix* | *Astragalus mongholicus* Bunge |
| 45 | Longyanrou | Sapindaceae | *Dimocarpus longan* | *Dimocarpus longan* Lour. |
| 46 | Yuanzhi | Polygalaceae | *Polygalae Radix* | *Polygala tenuifolia* Willd.  *Polygala sibirica* L. |
| 47 | Shengdihuang | Scrophulariaceae | *Rehmanniae Radix* | *Rehmannia glutinosa* (Gaertn.) Libosch. ex DC. |
| 48 | Tiandong | Liliaceae | *Asparagi Radix* | *Asparagus cochinchinensis* (Lour.) Merr. |
| 49 | Danshen | Labiatae | *Salviae Miltiorrhizae Ridix Et Rhizoma* | *Salvia miltiorrhiza* Bunge |
| 51 | Zhusha | — | *Cinnabaris* | Cinnabar |
| 52 | Zhike | Rutaceae | *Aurantii Fructus* | *Citrus* × *aurantium* f. *aurantium* |
| 53 | Zhizi | Rubiaceae | *Gardeniae Fructus* | *Plumeria rubra* L. |

**Note: The Latin names of TCM by Chinese Pharmacopoeia (2020 Edition). The source species family names, Latin names from MPNS (http://mpns.kew.org).The plant name has been checked with http://www.worldfloraonline.org. Date of access: 6 June 2026.**
